# Biomechanics of Transcatheter Aortic Valve Implant

**DOI:** 10.3390/bioengineering9070299

**Published:** 2022-07-04

**Authors:** Francesco Nappi, Sanjeet Singh Avtaar Singh, Pierluigi Nappi, Antonio Fiore

**Affiliations:** 1 Department of Cardiac Surgery, Centre Cardiologique du Nord, 93200 Saint-Denis, France; 2 Department of Cardiothoracic Surgery, Aberdeen Royal Infirmary, Aberdeen AB25 2ZN, UK; sanjeetsinghtoor@gmail.com; 3 Department of Clinical and Experimental Medicine, University of Messina, 98122 Messina, Italy; nappi.pierluigi@gmail.com; 4 Department of Cardiac Surgery, Hôpitaux Universitaires Henri Mondor, Assistance Publique-Hôpitaux de Paris, 94000 Creteil, France; antonio.fiore@aphp.fr

**Keywords:** transcatheter aortic valve implantation, surgical aortic valve replacement, structural valve degeneration, transcatheter heart valves

## Abstract

Transcatheter aortic valve implantation (TAVI) has grown exponentially within the cardiology and cardiac surgical spheres. It has now become a routine approach for treating aortic stenosis. Several concerns have been raised about TAVI in comparison to conventional surgical aortic valve replacement (SAVR). The primary concerns regard the longevity of the valves. Several factors have been identified which may predict poor outcomes following TAVI. To this end, the lesser-used finite element analysis (FEA) was used to quantify the properties of calcifications which affect TAVI valves. This method can also be used in conjunction with other integrated software to ascertain the functionality of these valves. Other imaging modalities such as multi-detector row computed tomography (MDCT) are now widely available, which can accurately size aortic valve annuli. This may help reduce the incidence of paravalvular leaks and regurgitation which may necessitate further intervention. Structural valve degeneration (SVD) remains a key factor, with varying results from current studies. The true incidence of SVD in TAVI compared to SAVR remains unclear due to the lack of long-term data. It is now widely accepted that both are part of the armamentarium and are not mutually exclusive. Decision making in terms of appropriate interventions should be undertaken via shared decision making involving heart teams.

## 1. Introduction, Search Strategy, and Selection Criteria

Transcatheter aortic valve implantation (TAVI) was first used by Cribier et al. 20 years ago [1]. Over the years, evidence has grown regarding the efficacy and safety of this novel modality, which has formed a major cornerstone in the treatment of structural heart disease. These minimally invasive procedures restore valve functionality in patients with calcific aortic valve stenosis (AVS) and have become routine approaches [2,3,4,5,6,7,8,9,10,11,12,13,14,15,16,17,18]. TAVI is recommended for symptomatic patients with severe AS who are 65 to 80 years of age and have no anatomic contraindications to the use of transcatheter aortic valve implantation via transfemoral access. TAVI is considered an adequate treatment option as an alternative to standard surgical aortic valve replacement (SAVR) after shared decision making, weighing the balance between expected patient longevity and valve durability [19,20,21,22,23,24,25]. Evidence suggested that TAVI (compared to standard medical and surgical options) had lower associated rates of death from any cause. Mid- and long-term follow-ups provided no evidence of restenosis or prosthesis dysfunction [6,9,10,11,18,26,27,28,29,30]. Moreover, recent randomized clinical trials (RCTs), meta-analyses, and propensity score analyses, confirming registry reports, revealed satisfactory outcomes of TAVI in terms of feasibility, long-term hemodynamics, and functional improvement [12,14,27,31,32,33,34]. However, the first and second generations of implanted transcatheter heart valves (THVs) had high related percentages of moderate to severe perivalvular aortic regurgitation [35], which is evidence that highlights the causes that determine one of the frequent complications associated with TAVI, which confers an increased rate of mortality [36]. During repeated follow-ups, the emerging data raised concerns about the incomplete apposition of prostheses related to calcification or annular eccentricity [37], the undersizing of the device, and the incorrect positioning of the valve, thus identifying the most common determinants of paravalvular aortic regurgitation [38]. 

Based on these observations, the criteria that are of utmost importance to avoid complications are the appropriate determination of the size of the annulus, the correct evaluation of the calcifications, and adequate sizing of the prosthetic valve. Pre-operative planning with biomechanical assessments should be completed for patients for whom TAVI is recommended, as suggested by international guidelines and by standardized endpoint definitions for transcatheter aortic valve implantation, dictated in the Valve Academic Research Consortium-2 (VARC-2) consensus document [19,20,38].

Finite element analysis using computational biomodeling is a crucial method used to obtain valuable measurements regarding complicated real-world systems which would otherwise be impossible to directly determine. Today, several studies have applied FEAs to the design of medical devices or to the analysis of mechanical processes integrated into the biological system in order to calculate stresses and investigate potential failure modes and locations. Finite element (FE) models require accurate 3D (3D) geometry in the zero-stress state, material properties, and physiological loading conditions [39,40,41,42,43,44,45,46,47,48,49,50,51,52,53,54,55,56,57,58,59,60,61,62,63,64,65,66,67,68,69,70,71,72,73].

To encourage a wider diffusion of TAVI, and to provide a guide for clinicians, we discuss the current evidence basis for the use of transcatheter heart valve implantation and review related articles focused on computational biomodelling aimed at predicting the failure of transcatheter heart valve therapy for the treatment of structural heart disease [19,20,21,22,23,24,25,26,27,28,29,30,31,32,33,34,35,36,37,38,39,40,41,42,43,44,45,46,47,48,49,50,51,52]. 

Given that TAVR has shown similar results to the standard surgical procedure in intermediate-risk patients and TAVI is now widely used as an approach in patients at risk, the main concerns are the long-term durability of TAVI and the risk of thrombosis. In fact, the evidence has amply reported the duration of surgical bioprosthesis, the low risk of developing thromboembolism, and the absence of problems related to valve deployment after the standard surgical approach [43,53,54,55,56,57,58,59,60,61,62,63,64,65,66,67,68,69,70,71,72]. We know that from a pathological point of view, bioprosthetic degeneration involves leaflet cusp calcification and stiffening associated with leaflet tearing. In addition, areas of increased stress are strictly related to regions of calcific degeneration or leaflet tearing. This process led to an increased risk of reoperation [43,53,54,55,56,57,58,59,60]. 

We searched MEDLINE, Embase, and the Cochrane Library using the search terms “aortic valve stenosis” or “aortic valve operation” together with “transcatheter aortic valve implant”, “transcatheter aortic valve replacement”, “standard surgical aortic valve replacement”, “computational modelling”, “finite element analysis”, “aortic valve surgery”, “transcatheter heart valve” or “valve thrombosis”, and “ structural valve degeneration”. We selected publications primarily within the past 20 years; however, we did not exclude widely referenced and highly regarded older publications. Recommended bioengineering articles were cited to provide readers with further details and background references.

**Figure 1:**
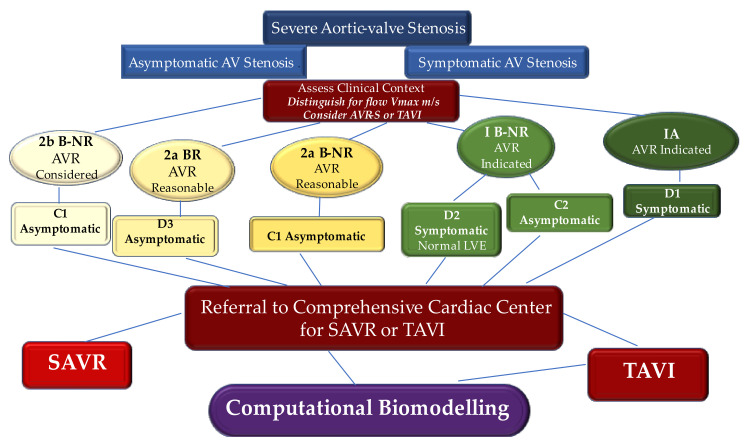
Decision Tree for treatment of severe AVS based on international guidelines and the VARC-2 consensus document. Recommendations from the 2020 international guidelines (ACC/AHA/ESC) for the treatment of patients with valvular heart disease. Clinical factors and imaging findings are shown in green and yellow boxes as well as AVR recommendations according to Class (Strength) of Recommendation and Level (Quality) of Evidence. Treatment recommendations are shown in red boxes. 1A, 1B-NR, 2aB-NR, 2a B-R, and 2b NR are the CORs which indicate the strength of recommendation, including the estimated magnitude and assurance of advantage in relation to risk. The LOE rates the quality of scientific evidence supporting the intervention based on the type, quantity, and consistency of data from clinical trials and other sources. Computational biomodelling is a suitable method for a predictive evaluation of TAVI performance. Abbreviations: AVS, aortic valve stenosis; COR, class of recommendation; LOE, level of evidence; SAVR, standard aortic valve replacement; HF, heart failure; LVF, left ventricular function; TAVI, transcatheter aortic valve implant, VARC, Valve Academic Research Consortium.

We broadly address the use of computational biomodelling to further appreciate complex mechanical processes regulating the workings of these new devices for aortic root implantation. Using advanced computational tools that integrate patient-specific information, it is thereby possible to obtain accurate modeling of the self- and balloon-expandable devices used to treat severe aortic valve stenosis. We propose an evidence-based algorithm for the choice of TAVI [19,20,21,22,23,24,25,26,27,28,29,30,31,32,33,34,35,36,37,38,39,40,41,42,43,44,45,46,47,48,49,50,51,52,53,54,55,56,57,58,59,60,61,62,63,64,65,66,67,68,69,70,71,72] (Figure 1).

## 2. Engineering to Study the Features of Implanted Transcatheter Heart Valve

Transcatheter aortic valve implantation is becoming the prime destination on the road map for translational research since its first ideation and use in pediatric cardiac surgery to circumvent the complication of reopening the sternum and reoperation [53]. Using the finite element analysis (FEA) methodology, we marked the crucial differences between the biomechanics of the aorta and pulmonary artery [54,55]. We performed a tensile test in the native pulmonary artery and native aorta. Evidence suggested that tissue’s response to stressors of the pulmonary valve leaflets caused stiffer behavior than the aortic valve, and decreased deformation for applied loads as high as 80 kPa (600 mmHg) was recorded. Importantly, the biomechanics of the valve annulus displayed less deformable structures of the root, suggesting that the weaker points of the PA were present in the free walls of the pulmonary artery (PA) distal to the valve. The aortic root suitably accommodated increasing hemodynamic loads without meaningful deformation. Again, the differential analysis performed on samples cut longitudinally and circumferentially revealed different behavior for both the aorta and pulmonary artery. The circumferential strength of the PA was greater than the aortic one, while similar properties in the longitudinal direction were comparable. Our results suggested that the PA may exhibit a consensual increase in stress and strain in both directions, while the aorta revealed better adaptability in the longitudinal direction and a steeper curve in the circumferential response, potentially suggesting the non-aneurysmatic tendency of the pulmonary artery root compared to the aorta [54].

The innovative use of FEA for research in cardiovascular science related to the mitral valve, pulmonary artery, and aorta [41,42,43,50,51,52,56,57,58,59,60,61,62,63,64,65,66,67] can provide an understanding of structural changes in biological systems such as degenerative processes in leaflet and vessel wall stresses, thereby preventing procedural failures. The distinct measurement of biomechanical stress resulted in different applicability in studies such as those investigating leaflet stresses related to the geometry of stented porcine and bovine pericardium xenografts [57] or examining stresses in the aortic root and calcified aortic valve aimed to prevent the risk of rupture [41,43,44,59,60,68]. Recently, the benefits associated with the use of FEA applied to TAVI were established in a landmark paper by Xuan et al. The investigators thoroughly evaluated TAVI with leaflets, stents, polyethylene terephthalate, and sutures to predict the mechanism leading to the structural valve degeneration of THV devices [56].

### 2.1. Confluence of Engineering and Medical Sciences

Finite element analysis is a discipline of the geometric algorithmic prediction of stress and the evaluation of deformation coefficients in complex structures through a complex system of predictable mathematical calculation applied to well-divided small geometric areas [68]. We have learned that from its first applications in the field of cardiac surgery, which date back about twenty years, the use of FEA has developed slowly despite the possible achievement of substantial progress. Since its introductory applications, the FEA methodology has been noted for its limited applicability in clinical practice. This ‘distrust’ is pertinent in surgical disciplines, which are based on clinical evidence, as the Finite Element Analysis investigation offers their field of research speculative data without correlated clinical evidence [40,41,42,43,54,55,59,60,61,67].

Before the paradigm shift that radically changed the treatment of symptomatic calcific aortic stenosis, clinical and experimental studies produced scientific evidence without the use of FEA. Easier, more understandable methodologies and probably more reliable ones have been used to test hypotheses and prove theses. The revolutionary technology of the novel method that makes up the most advanced platforms for the treatment of structural heart diseases meant that SAVR had given way to the advent of TAVI. Rapid technological advancements have made it possible to obtain three generations of balloon-expandable devices in a span of 6 years and has given new impetus to FEA [2,3,4,5,6,7,8,9,10,11,12,13,14,15,16,17,18].

In this context, the findings of Smuts et al. aided the development of new concepts for different percutaneous aortic leaflet geometries [69]. Instead, Wang et al. [43] and Sun et al. [70] studied the post-operative behavior of TAVI from a mechanical and hemodynamic point of view. A crucial advancement in the application of FEAs was offered by Capelli et al. [45], who effectively analyzed the feasibility of TAVI in morphological conditions and considered borderline cases for the percutaneous approach, paving the way for the treatment of failed bioprosthetic aortic valves with the use of TAVI. 

A patient-specific simulation based on FEA that takes into account all procedures and has the potential to produce post-operative prosthesis simulations, by means of inclusion in the analysis of biological valve needlework in metal structures, was reported by our group in a landmark paper almost 10 years ago [71]. We subsequently reported evidence by comparing the post-operative medical data with the biomechanical investigation method. Recently, we developed a systematic TAVI simulation approach, tailored for clinical practice, for patients receiving both a self-expandable Medtronic Corevalve (Medtronic, Minneapolis, MN, USA) and a balloon-expandable SAPIEN (Edwards Lifesciences, Irvine, CA, USA). Studies based on the analysis of the pre-operative medical imaging of patients who have undergone TAVI are of particular interest [39,40,41,50,51,52]. The final goal derived from these studies is to predict the post-operative performance of the prosthesis with respect to the specific anatomical characteristics and potential complications such as structural/non-structural valve degeneration and thrombosis [56].

Likewise, the new evidence emerging from these studies strengthened previous evidence on the potentially high levels of stress to which devices for THV implantation are subjected. Previous studies have revealed, both in a static or boundary conditions as well as during fatigue stress simulations that in individuals who are managed with the THV procedure, the predictable duration of TAV1 may be shorter than those who received a surgically implanted aortic bioprosthesis. This evidence confirms that leaflet deformation and stresses are significantly higher in TAVI, especially near commissures and along stent attachments [57,72].

### 2.2. Medical Image Processing

Biomechanical simulations using FEA analysis, starting from pre-clinical evaluations, have offered an original contribution as an advanced tool for clinical support for the following reasons. First, the aortic valve model is complete, including both the aortic sinuses, and the native valve leaflet as well as the material model considered are calibrated on human data. Second, the calcified plaque is included in the model, and it is based on the image recording. Finally, the geometry of the prosthetic stent is very precise, obtained from micro-tomography (micro-CT) reconstruction [39,40,41,50,51,52].

Another substantial advantage that makes this analysis reliable is represented by the possibility of obtaining post-operative data collected by physicians for the follow-up of individuals. These data are used for comparison with the numerical results obtained by the FEAs, with the ultimate goal of evaluating the capabilities of the proposed simulations to predict procedural outcomes [40,50].

Concerns related to validating TAVI simulations are crucial as it can usually be difficult to obtain good-quality post-operative data and images from standard post-operative procedures. Another point of divergence concerns post-operative CT control, which is sometimes excluded from routine protocols for TAVI because these patients are often frail, and it is not recommended to overload the kidneys with additional doses of contrast and high doses of radiation should be avoided in patients who are often in critical condition. Instead, evaluations on the outcome of the procedure are offered by intraoperative CT scans as well as by follow-up echotomography [73,74,75].

**Figure 2:**
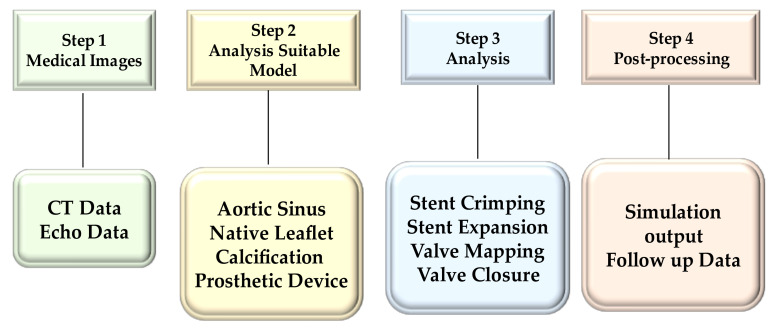
The computational framework aimed to simulate the implantation of TAVI. Four sections from Steps 1 to 4 of the worked-out modeling strategy are identified. The extrapolated images (ECHO and CT) allow biomodelling on which to perform the simulations to be established. The data obtained from the simulations are compared to the data that emerged in the follow-up. Abbreviations; CT; computed tomography.

The computational framework adopted to simulate the implantation of TAVI includes four main phases, which are processing of the medical images, the creation of models suitable for analysis, the performance of the required analysis permitting the integration of the clinical procedure, and finally, the post-processing of the simulation results and subsequent comparison with the follow-up data [39,40,41,44,50,51,52] (Figure 2).

Morganti et al. worked on a biomechanical simulation model for TAVI starting from a standardized approach to scan the main parameters with cardiac CT. Pre-operative examinations were obtained using a dual-source computed tomography scanner (Somatom Definition, Siemens Healthcare, Forchheim, Germany). The investigators achieved contrast-enhanced images using iodinated contrast medium which was injected as follows: scan direction, cranio-caudal; slice thickness, 0.6 mm; spiral pitch factor, 0.2; tube voltage, 120 kV [40,41].

**Figure 3:**
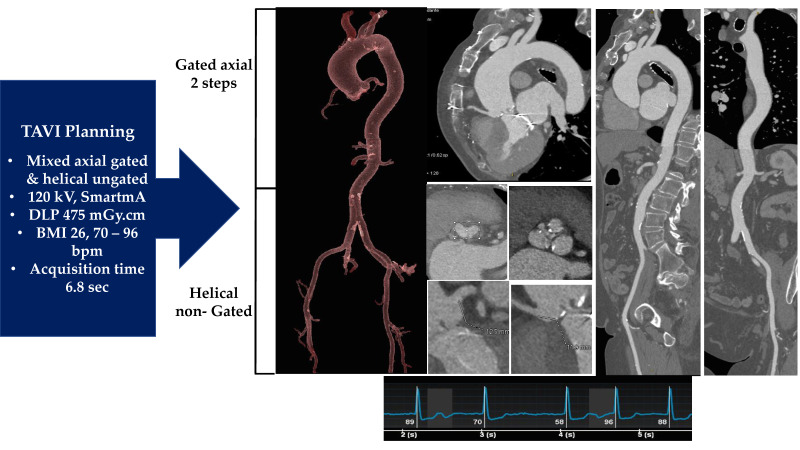
The 3D CT scan protocol for patients receiving TAVI. It is designed to ensure all steps of the transcatheter procedure. The figure reports an example in which the exam was conducted in 6.8 s with only 640 mGy.cm. Precise planning to support the intervention was conferred. Abbreviations; DLP, Dose Length Product; mGy, microgray.

Our group developed a reliable protocol to ensure the quality of the CT images, which must subsequently be processed using FEA [39,50,51,52] (Figure 3).

**Figure 4:**
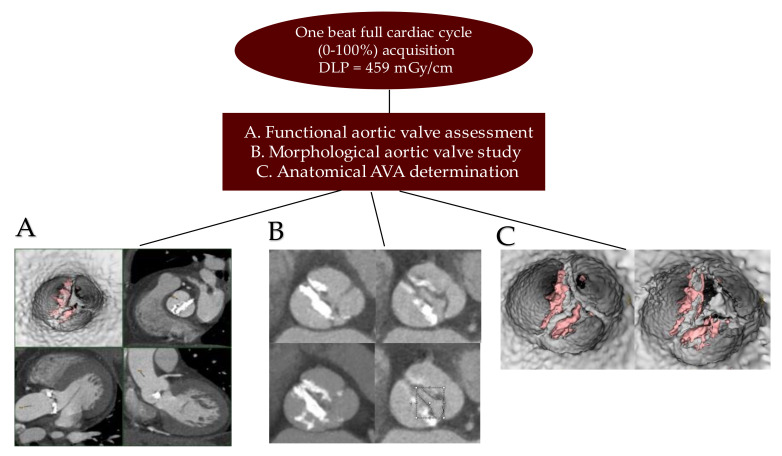
DICOM from a 3D CT scan serves to extract the RAW data that allow definition of functional aortic valve assessment (**A**), morphological aortic valve features (**B**), and anatomical aortic valve characteristics (**C**); abbreviations in other figures.

With a complete cardiac cycle in one beat (0–100%) and with the acquisition of a Dose Length Product (DLP) equal to 459 microgray (mGy)/cm, we offered an optimal image quality to be processed for biomechanics. This allowed the functional evaluation of the aortic valve, the morphological study of the aortic valve, and the anatomical determination of the AVS [39] (Figure 4).

Scientific reports that describe image analysis using established theoretical approaches have provided solid answers on the active contour segmentation process, which has experienced robust implementation. Despite the existence of powerful segmentation methods, the needs of clinical research have continued to be met, to a large extent, using manual slice-by-slice tracking. The landmark study of Yushkevich et al., performed in the context of a neuroimaging study of childhood autism, bridged the gap between methodological advances and routine clinical practice. The investigators developed a revolutionary open-source application called ITK-SNAP. This application aims to make the segmentation of level sets easily accessible to a wide range of users, including those with little or no mathematical skills. SNAP proved to be a reliable and efficient application compared to manual tracking [76].

**Figure 5:**
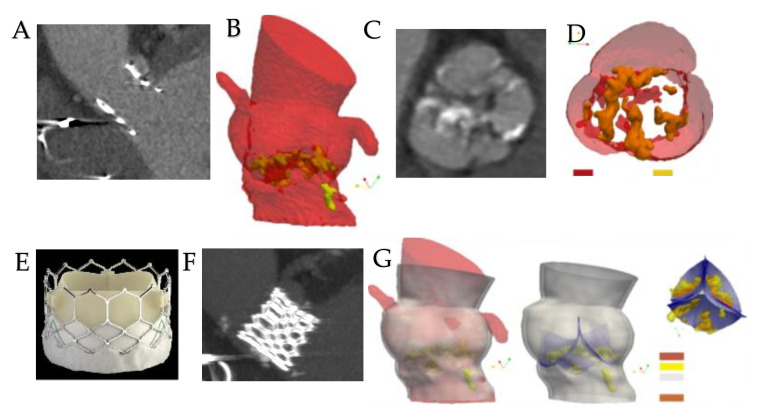
**Up**: With the use of ITK-Snapv 2.4 the data extracted from the CT images, (**A**,**C**) are processed to highlight the images of the aortic lumen (**B**, red) and calcium deposits (**D**, yellow/orange). **Down**: A first generation of balloon-expandable TAVI Sapien (**E**; Edwards Lifesciences, Irvine, CA, USA) is used to treat severe AVS (**F**). **G**: Aortic lumen morphology as well as the calcium conglomerates are extracted with the use of STL files. **Left**: The lumen of the aortic root (red) and the calcifications (yellow) are superimposed to the aortic wall model (gray). **Center**: The enclosed native leaflets (blue mesh) correspond perfectly to the real leaflets with calcifications obtained by processing CT images (**A**,**C**). **Right**: The top view is shown. Abbreviations: AVS, aortic valve stenosis; CT, computed tomography; STL, stereolithographic; TAVI, transcatheter aortic valve implantation.

Therefore, the most common method of obtaining a reliable model from CT data sets is their processing using ITK-Snapv2.4, as described by Yushkevich et al. [76]. Specifically, a confined region of interest, such as that represented by the aortic root, which is composed of the left ventricular outflow at the sinotubular junction, is extracted from the entire reconstructed body by exploiting the contrast enhancement, nibbling, and segmentation capabilities of the software. Again, the effectiveness of the TK-Snapv2.4 is highlighted using different Hounsfield unit thresholds, through which it is possible to distinguish the calcium agglomerates of the surrounding healthy tissue and evaluate it at intervals of both position and size. Once the segmented regions have been extracted, it is possible to export the aortic lumen morphology, as well as the calcium deposits like stereolithographic (STL) files [39,40,41,50,51,52] (Figure 5).

### 2.3. Analysis Suitable Model

A crucial step concerns the procedure to obtain suitable analysis models both for the native aortic valve, including calcifications affecting the leaflets along with the aortic wall, and for the prosthetic device. 

#### 2.3.1. Native Aortic Valve Model

In the native aortic valve model, different investigators reported that once the STL file containing the characteristics of the aortic root is obtained, it can be processed and implemented in Matlab (The Math works Inc., Natick, MA, USA). The latter serves as an effective system for defining a set of splines, similar to the cross-sectional profile of the aortic lumen. In this way, the curves obtained are used to automatically generate a volume model of the aortic root wall. 

A finite element analysis setup is ensured by importing the model that has been processed in commonly used software such as Abaqus CAE (Simulia, Dassáult Systems, Providence, RI, USA) or alternatively HyperMesh (Altair Engineering, Troy, MI, USA). The latter may be used in association with GeoMagic Design (3DSystems, Rock Hill, SC, USA), a computer-aided design software, to purify and generate 3D geometric volume with accurate size and thickness at zero stress [39,40,41,50,51,52,53,56,71,77].

Several studies demonstrated that the geometric model of the aortic root obtained by processing the STL file represents the fundamental starting point for performing the finite element analysis of TAVI. Antiga et al. created the Vascular Modeling Toolkit (VMTK). This modeling framework was designed for patient-specific computational hemodynamics to be performed in the context of large-scale studies. The use of Vascular Modeling Toolkit exploits the combination of image processing geometric analysis and mesh generation techniques and stresses full automation and high-level interaction. Importantly, image segmentation is performed using inferred deformable models and by exploiting the advantage of a different approach for the selective initialization of vascular branches, as well as of a strategy for the segmentation of small vessels. Again, the advantage of using the Vascular Modeling Toolkit is the solid definition of center lines which provides substantial geometric criteria for the automation of surface editing and mesh generation [77,78].

Several investigators reported good results by processing STL files of calcifications using the Vascular Modeling Toolkit to extract a regular tetrahedral mesh [39,40,41,50,51,52,53,56,71,77,78]. Likewise, an efficient, robust procedure for the mesh generation leading to high-quality computational meshes includes the open-source Gmsh software [79] and the alternative framework described by Dillard et al., in which the entire image-based modeling process is performed on a Cartesian domain where the image is fixed within the domain as an implicit surface [80]. Gmsh software can generate different types of meshes including isotropic tetrahedral meshes, anisotropic tetrahedral meshes, and mixed hexahedral/tetrahedral meshes. In addition, Gmsh software had the crucial advantage of generating multiple-layered arterial walls with variable thicknesses. Alternatively, the structure developed by Dillard et al. gets around the need to generate surface meshes that have to adapt to complex geometries and the subsequent need to generate flow meshes adapted to the body. The three determining factors are identified as Cartesian mesh pruning, local mesh refinement, and massive parallelization, which are crucial to providing computational efficiency. The efficacy of the framework described by Dillard et al. lies in the full picture analysis, which revealed two 3D image reconstructions of geometrically dissimilar intracranial aneurysms which require computed flow calculations [80].

The finite element mesh generated with this procedure is effective for both reproduced aortic wall and native valve leaflets in obtaining a complete and realistic model to perform the simulations at the same time. Morganti et al. suggested that to include the native geometry of the leaflets, the first step consists of identifying nine reference points: six of them refer to the commissural extremes, while the others correspond to the center of the attachment of the basal leaflets. We recently adopted this method in a study comparing two different biomechanical features involving the two different TAVI device models, the self-expanding Medtronic CoreValve and the balloon-expandable Edwards SAPIEN [40,41]. Of note, Xuan et al. also revealed that stent and leaflet surfaces were combined using suture lines as a reference point for leaflet orientation [56].

It is important to highlight that the use of the aforementioned reference points offers the possibility of defining individual planes that can guide the distribution of the entire model of the aortic root, which ultimately serves to reproduce both the extraction of the leaflet commissures and the attachment lines [40,41,50,51]. The use of ultrasound is important to measure the length of the free margins, which appear as a circular arc. Determining the perimeter of the leaflets leads to the construction of the leaflet surface in the open configuration [40].

The modeling of the aortic wall is meshed with the use of a variable number of tetrahedral elements that take into account both the healthy part and the portion occupied by calcium conglomerates. Morganti reported a number between 235,558 and 265,976 tetrahedral elements for the healthy region of the aortic root, while the leaflet was discretized using a number between 3212 and 3258 shell elements for the healthy part. In cases where calcium agglomerates were present, the leaflets were discretized with reduced integration for healthy tissue. The discretization for the occurrence of calcified plaques ranged from 342 to 427 shell elements [40,41].

Xuan et al. worked to determine stent and leaflet stresses in a 26 mm first-generation balloon-expandable transcatheter aortic valve. The investigators imported the refined geometries of leaflets, stent, and polyethylene terephthalate into HyperMesh (Altair Engineering, Troy, MI, USA) to generate TAV mesh with 46,443 total elements. Their study did not require adjunctive discretization for the presence of calcified plaques located in the aortic wall and leaflets because the simulation was not performed in the aortic root and leaflets cluttered by calcifications [56]. 

Bianchi et al., in a comparison study between Sapien 3 and CoreValve, squeezed out the sinuses of Valsalva in Abaqus CAE, while the calcification deposits were processed in MATLAB and subsequently assembled in the AR. In a previous report, Bianchi et al. [47] incorporated calcifications in soft tissues to better mimic the morphology of the stenosis. The investigators finally re-meshed the aortic root with tetrahedral elements in Ansys Fluent Meshing to ensure mesh continuity at the interface between the sinus and the leaflets and between calcifications and surrounding soft tissues. The mesh size was approximately 1.4 million for SAPIEN cases and 2.5 million for CoreValve cases, as more of the ascending aorta were required for deployment.

In cases of biomechanical evaluations used to compare prosthetic devices, post-operative configuration, and performance, simplified St. Venant-Kirchhoff properties can be used to model native aortic tissue, leaflets, and calcifications. Several investigators used Young’s modulus for the aortic root, leaflets, and calcifications (E, Poisson’s ratio ν, and density ρ) [40,81]. Xiong et al. used Young’s modulus for the native leaflet, and they used such a value to model the bovine pericardium aortic leaflet [81]. Stradins et al. reported that the same value of 8 MPa approximates the stiffer (i.e., circumferential) non-linear behavior of the human aortic valve. It is important to underline that considering the stiffer curve is reasonable given the greater stiffness recorded in aortic valve stenosis, which have stiffer tissues than the average patient [82].

#### 2.3.2. Prosthesis Model and Material Model

Although several devices for TAVI have been described over the 20 years, [39] the two devices used in a large number of patients in clinical practice include the Medtronic Core Valve and the Edwards Lifesciences SAPIEN. While the CoreValve is self-expanding, the Edwards SAPIEN valve is primarily produced of three flexible biological leaflets sutured into an expandable balloon stent.

Several studies reported computational biomodelling studies of SAPIEN first-generation, [42,56] XT [40], and the last SAPIEN 3 [50] prosthesis starting from 3D CT scans of patients who underwent TAVI. Likewise, the same investigators worked on the computational biomodelling studies of CoreValve Medtronic [41,42,50,52].

For example, in two independent works, Morganti et al. [40] and Nappi et al. [50] obtained a faithful geometrical model of SAPIEN XT 26 mm and of SAPIEN 3 using a high-resolution micro-CT scan (Skyscan 1172 with a resolution of 0.17 micron). These stent models were achieved using 84,435 solid elements. Xuan et al. [56] obtained a fully expanded first-generation Sapien valve (26 mm) which was conceived under 0 mm Hg pressure with a desktop cone-beam micro-CT scanner (microCT-40; Scanco MedicalAG, Baseldorf, Switzerland) in different orientations and intensities to discriminate stent and leaflet geometries. The refined geometries of leaflets, stents, and polyethylene terephthalate were then imported into HyperMesh (Altair Engineering, Troy, MI, USA) to produce TAV mesh with the use of 46,443 total elements [56].

Generally, the material model for the native aortic tissue is presupposed to be homogeneous and isotropic, as described by Capelli et al. [45] and Gnyaneshwar et al. [83]. Selvadurai [84] and Yeoh et al. [85] hypothesized the use of an incompressible reduced polynomial form aimed at reproducing the material behavior and indicating it as reduced polynomial strain energy, taking into account the material parameters of the deviatoric strain invariant and the deviatoric stretches. 

Morganti et al. [40], in the computational modeling of SAPIEN XT, with regard to the material model, chose a sixth-order polynomial form, finding an unknown material constant. The investigators took as reference for the aortic leaflets and the Valsalva sinuses the data that emerged from the studies by Martins et al. [72] and Stradins et al. [82]. These data were integrated with those produced by Auricchio et al. to obtain the final characteristics of the material models. In particular, with regard to the aortic wall and the native valve leaflets, it was assumed that these had a uniform thickness of 2.5 and 0.5 mm, respectively. In observations of the evidence reported by Capelli et al. [45], for calcifications, an elastic modulus of 10 MPa; a Poisson ratio of 0.35; and a density of 2000 kg/m^3^ were assumed. Again, as for the Von Mises plasticity model with isotropic hardening, Morganti et al. assumed 233 GPa as Young’s modulus; 0.35 as the Poisson coefficient; 414 MPa as yield stress; 933 MPa as ultimate stress; and 45% from deformation at the break [40,41]. 

The computational model that evaluates the prosthetic valve leaflets of the SAPIEN device must consider the different factors concerning the constitutive characteristics of bovine pericardium after the fixation process. The leaflets were modeled as an isotropic material and, in particular, an elastic modulus of 8 MPa, a Poisson coefficient of 0.45, and a density of 1100 kg/m^3^ were used following the evidence reported by Xiong et al. The prosthetic valve was meshed with 6000 quadrilateral shell elements, while a uniform thickness of 0.4 mm was considered [40,81,86,87,88,89].

#### 2.3.3. Finite Element Analyses

Finite element analysis is a crucial step of computational biomodelling to be applied to the TAVI procedure for biomechanical evaluation. Since TAVI is a complex procedure that is divided into several phases, the simulation must respect rigid steps to be reliable, which are stent crimping/deployment and valve mapping/closure.

**Figure 6:**
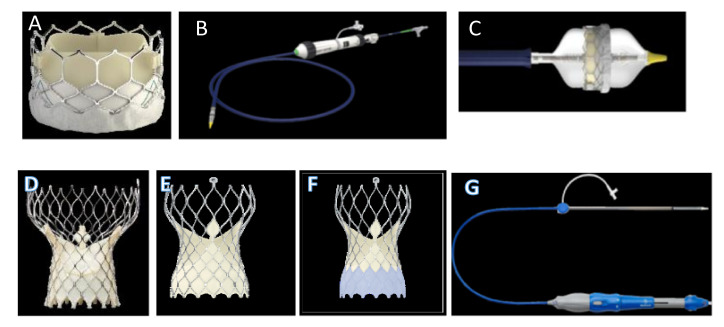

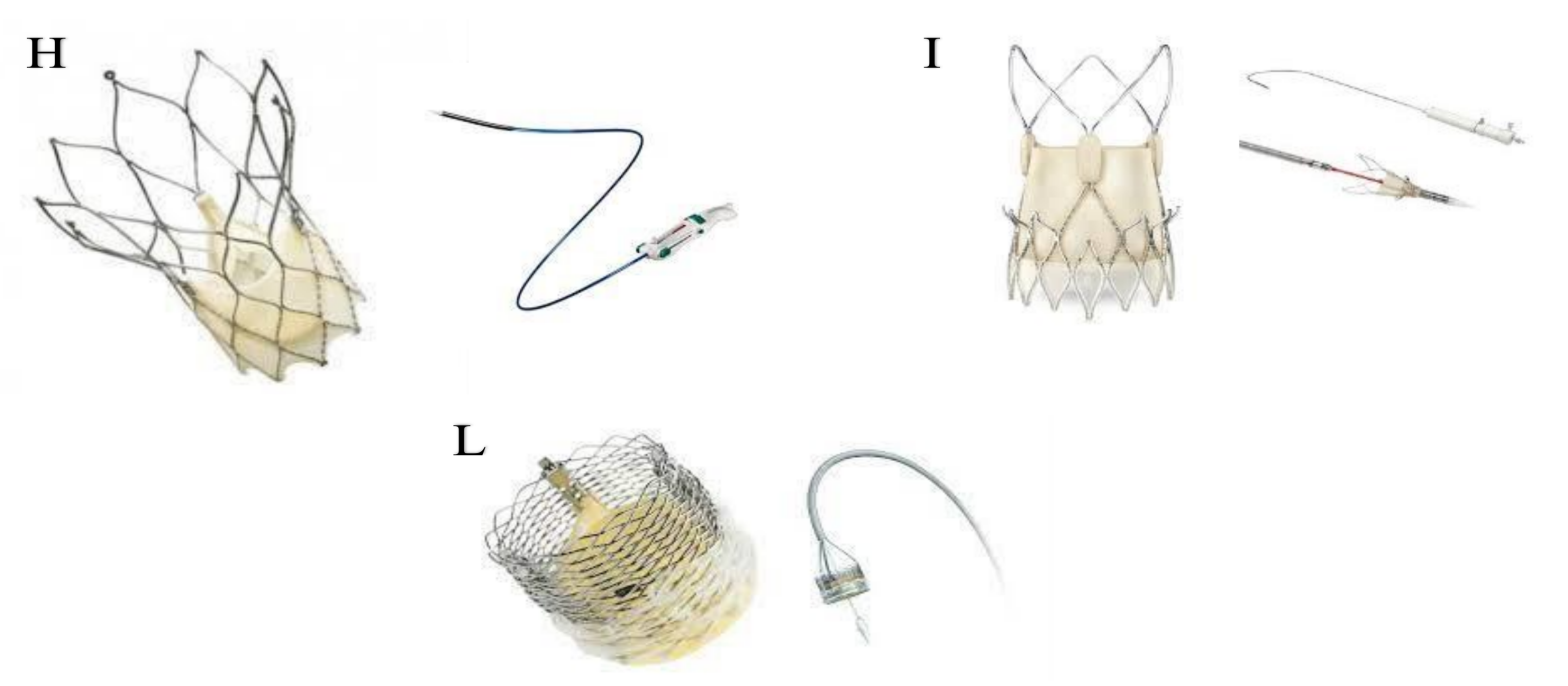
Balloon-expandable THV. (**A**–**C**) The SAPIEN 3 balloon-expandable device is constituted by a cobalt–chromium alloy frame valve with bovine pericardium leaflets. The device is available on the market in the following sizes: 20 mm, 23 mm, 26 mm, and 29 mm (**A**). The Commander Delivery System is 14 F expandable introducer sheath compatible with 20–26 mm valves and 16 F expandable introducer sheath compatible with 29 mm valves (**B**,**C**). (**D**–**G**) Self-expandable THV. The bioprosthesis is manufactured by suturing 3 valve leaflets and a skirt, made from a single layer of the porcine pericardium, onto a self-expanding, multi-level, radiopaque frame made of Nitinol (**D**–**F**). CoreValve (**D**), Evolut R (**E**), and Evolut PRO (**F**) in the following sizes: 20 mm, 23 mm, 26 mm, and 29 mm. ©. The loading system. The outer diameter of the catheter is 15 Fr (AccuTrak™ stability layer) and 12 Fr, and the outer diameter of the valve capsule is 18 Fr. The catheter can be used for femoral, subclavian/axillary, or ascending aortic (direct aortic) access sites. (**H**) The Portico re-sheathable transcatheter aortic valve system (Abbott Structural Heart, St Paul, MN, USA). (**I**) The ACURATE neo (Boston Scientific, Marlborough, MA, USA) self-expanding THV. (**L**) Lotus mechanically expanded valve (Lotus Valve System (MEV; Boston Scientific Corp., Natick, MA, USA) (**H**): Portico valve is designed with large, open cells and intra-annular leaflet placement to preserve flow and access to the coronary arteries after deployment The Portico valve was delivered by a flexible, first-generation Portico Delivery system, which had an 18 F outer diameter for the small valves (23 and 25 mm) and a 19 F outer diameter for the larger valves (27 and 29 mm). (**I**): the ACURATE neo bioprosthesis consists of a self-expanding nitinol frame with three porcine pericardial leaflets and a stent body with an outer and inner pericardial skirt. (**L**): The MEV is constituted by 3 bovine pericardial tissue valve leaflets and a braided nitinol frame with a polycarbonate-based urethane adaptive seal. λ From Willson AB et al., transcatheter aortic valve replacement with the St. Jude Medical Portico valve: first-inhuman experience. *J. Am. Coll. Cardiol.* 2012; 60: 581–86; † From Mollmann H, *EuroIntervention* 2013; 9 (suppl): S107–10. from Meredith IT et al. Boston Scientific Lotus valve. *EuroIntervention*. 2012; 8 (suppl Q): Q70–Q74. Abbreviation. MEV = mechanically expanded valve. THV = transcatheter heart valve.

In the first stage, the prosthetic model is crimped to obtain the catheter diameter, which was usually 24 French (8 mm) in the transapical approach. Subsequently, the prosthetic prosthesis expands inside the AR. In the aortic root, the device is expanded according to the two most widely used systems: the self- and the balloon-expandable method [3,8,90,91]. A third system is represented by mechanical expansion [92,93]. The transapical approach has been replaced by the transfemoral one, which is currently a more commonly adopted procedure and benefits from the use of small catheter sizes of 18–16 and 14 French [15,16,17] (Figure 6).

Again, all the numerical analyses are subject to non-linear concerns involving large deformation and contact. For this reason, many investigators used the Abaqus system (solver v6.10 or CAE) [40,41,42,46,50,51,52,56] to perform analyses on large deformations. Two points still need to be emphasized. First, quasi-static procedures were used, again assuming that inertial forces do not change the solution. Second, kinetic energy monitoring is crucial; kinetic energy is monitored to ensure that the ratio of kinetic energy to internal energy remains less than 10%.

For example, with regard to stent crimping and deployment evaluating the procedure for a 26 mm SAPIEN XT implanted with a transapical approach, the cylindrical surface is gradually crimped from an initial diameter of 28 mm to a final diameter of 8 mm [40]. The cylinder is meshed using 2250 four-node surface elements with decreased integration, and it is modeled as a rigid material with a density of 7000 kg/m^3^. In these cases, a frictionless contact must also be considered, which is generally defined between the crimp surface and the stent. After affixing the stent, its deformed configuration is then re-imported into Abaqus CAE, taking into consideration the tensional state resulting from the crimping analysis as the inceptive state. Conversely, to reproduce the stent expansion, it is important to keep in consideration that a pure and uniform radial displacement is gradually applied to the node of a rigid cylindrical surface. Note that if a balloon-expandable device is used, it is assumed that the cylindrical surface is represented by the wall of the expanding balloon. Finally, the rigid cylinder is expanded from an initial diameter of 6 mm to a final diameter of 26 mm. Another fundamental point to consider in the simulation is that during the expansion of the stent, the axis of the balloon always remains fixed. This hypothesis can be considered valid because it is observed through intraoperative angiographic control that shows negligible axis rotation and translation [40,41,42,46,50,51,52,56].

The second stage is constituted by valve mapping and closure, in which the prosthetics leaflet is delineated onto the embedded stent ensuring physiological pressure that is requested to revive the diastolic behavior of implanted THVs. The pivotal study of Auricchio et al. [71] offered a substantial contribution to reproducing the realistic features of the prosthetic device, thereby evaluating the post-operative performance of implanted THVs. The investigators realized that pre-computed shifts are assigned to the base of the valve and at the nodes of the commissures of the leaflets so as to obtain a complete configuration of the implanted prosthetic device [40,41,42,46,50,51,52,56].

**Figure 7:**
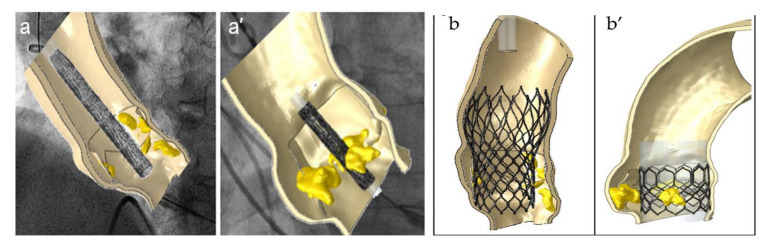
FEA simulations of TAVI in two investigated patients who showed post-operative thrombosis. The positioning (**a**,**a′**) and reopening (**b**,**b′**) of CoreValve and SAPIEN devices (left and right sides, respectively). Abbreviations in other figures.

By respecting these steps, it is possible to reproduce the post-operative diastolic features of both the balloon- and the self-expandable TAV within the patient-specific model of the aortic root. As reported by Wiggers et al., to simulate valve behavior at the end of the diastolic phase, uniform physiologic pressure needs to be applied to the prosthetic leaflet of the THV. Furthermore, a frictionless self-contact that is settled for the prosthetic valve must be considered [94] (Figure 7).

## 3. Insight on the Use of Biomechanical Evaluation to Predict Paravalvular Aortic Regurgitation

We have learned that the choice of the size and type of the prosthetic device is very important to avoid or at least reduce aortic regurgitation and/or other TAVI complications [35,37,95]. Detain et al. [35] and Delgado et al. [37] first independently reported that the occurrence of aortic regurgitation (AR) was related to incongruence between prosthesis and annulus. Since then, adequate annular sizing of the prosthesis has been considered essential to reduce paravalvular aortic regurgitation. Evidence that emerged from pivotal RCTs in patients who underwent THV implantation disclosed that very few TAVI candidates were supported with the anatomic and morphological study on the features of the aortic valve annulus to predict aortic regurgitation after device implantation [2,3,8].

Detain studied 74 patients who underwent TAVI with a balloon-expandable device with all-embracing echocardiographic examinations. The most favorable targets to disclose the occurrence of AR > or = 2/4 were greater patient height, larger annulus, and smaller cover index (all *p* < 0.002), while the ejection fraction, severity of stenosis, or prosthesis size were not indicative of AR-related events. Significantly, AR >2/4 was never displayed in patients with aortic annuli < 22 mm or with a cover index >8%. The increase in the ability to perform the procedure did not appear to have a statistically significant effect. Significant improvements were obtained from the first 20 cases in which the rate of AR > 2/4 was 40%, while in the last 54 AR > 2/4, it decreased to 15% (*p* = 0.02); however, the former versus the last procedure was an independent predictor for RA recurrence (odds ratio: 2.24; 95% confidence interval: 1.07 to 5.22, *p* = 0.03) [37]. One study reported that the use of the three-dimensional transesophageal planimetry of an aortic annulus proved that the ‘mismatch index’ for the 3D planimeter annulus area was the only independent predictor of significant aortic regurgitation (odds ratio: 10.614; 95% CI: 1.044–17.21; *p* = 0.04). Three-dimensional transesophageal planimetry improved the assessment of prosthesis/annulus incongruence and predicted the appearance of significant AR after TAVI as compared to the two-dimensional transesophageal approach [96].

MDCT is the type of imaging by which most of the evidence for the study of the aortic root is derived. In fact, four studies compared the anatomy of the aortic root with the size of the TAVI. Multi-detector row computed tomography was demonstrated to be a very effective tool to enable the accurate sizing of the aortic valve annulus and constitutes a valuable imaging implement to evaluate prosthesis location [95] and deployment after TAVI. Again, MDCT was a better predictor to detect a mismatch between prosthesis area and aortic annulus area [97] as compared to echocardiography, revealing pre- and post-procedure examination paravalvular aortic regurgitation (PAVR) ≥2+ at a rate of 20% at 1-month follow-up [98]. In one of the largest TAVI series published to date which checked patients pre- and post-operatively with MDCT, Katsanos et al. found that patients who were managed with TAVI and presented ≥2 mm difference between the maximum aortic annulus and nominal prosthesis diameters and depth of the frame into the left ventricular outflow tract of <2 mm were independently associated with PAVR ≥2+ occurrence. 

Madukauwa-David et al. [99] performed retrospective anatomical measurements post-TAVI in 109 patients with aortic stenosis obtained from the RESOLVE study using 4DCT scans. The investigators assessed the diameter of the aortic root at the level of the annulus, left ventricular outflow tract (LVOT), sinus of Valsalva, sinotubular junction (STJ), and ascending aorta. Again, the heights of the STJ and coronary arteries were determined. The major finding of the study proved that, by homogeneously distributing all aortic root dimensions in the cohort, they were susceptible to a statistically significant change between pre- and post-TAVR conditions (*p* < 0.01). The post-TAVR dimensions changed significantly from the peak systole to the end of diastole (*p* < 0.01). Regression models confirmed all measurements of the aortic root in terms of annular diameter, disclosing an excellent coefficient of determination (R2 > 0.95, *p* < 0.001). Researchers have suggested that there are significant differences between pre- and post-TAVR, affecting the anatomy of the aortic root both at the systolic peak and in the final diastolic part of the cardiac cycle. These findings can help select optimal THV device sizes that are appropriate to anatomical dimensions, as geometry varies greatly during the cardiac cycle [99].

Concerns related to the occurrence of PVAR and its worse evolution is due at least in part to the heterogeneity of the methods for assessing and quantifying PAVR. Moreover, the lack of consistency in the timing of such assessments leads to an obstacle to understanding its accurate prevalence, severity, and effect [35]. Choosing a correct prosthetic size does not seem to be the only way to avoid PVAR, but also, the complex original morphology of the aortic root and the location and size of the calcifications are crucial determinants to take into consideration. Again, the occurrence of solid annular calcium deposits that protrude more than 4 mm is a negative predictor of moderate to severe PVAR in patients undergoing TAVI. The morphology of calcium conglomerates is involved in the genesis of PVAR in relation to the size of the annular bulky calcification, which is another predictive factor, unlike adherent calcium, which has a “sealant“ effect [100].

Currently, the clinical benefits of computational analysis to guide the TAVI are not well established, and the approach represents the cornerstone of modern transcatheter heart valve therapy. The data that emerged in favor of computational analyses take into account the recipient of the transcatheter procedure and both the specific structure of the native aortic valve and an accurate evaluation of calcifications. These two parameters can offer a substantial contribution and, in association with dynamic fluid assessments, can support and guide device selection. 

Many investigators have confirmed the effectiveness of computational analyses by defining a reliable framework for reproducing the TAVI procedure and predicting any complications. As has been reported in several studies, the distribution of stress is characterized by concentrated spots of higher stress values that are recorded at the points of contact between the stent and the aortic wall [39,40,41,42,43,44,45,46,47,48,49,50,51,52,56]. We corroborated the evidence of Wang et al. [43], showing that the highest stress values were recorded in the aortic regions close to the calcifications both in self-expanded and balloon-expanded THV devices [50].

Similarly, Morganti et al. [40], in a computational analysis performed on a balloon-expandable device, found major stress levels in the region where the SAPIEN T-stent was most adherent to the aorta wall. Therefore, it has been suggested that higher stress values may be related to the greatest adhesion force between the aortic wall and the stent. Likewise, Eker et al. [101] firstly revealed that the creation of high levels of stress located in the annular region is not devoid of increased risk of aortic rupture, as a possible early complication of TAVI leading to cardiac tamponade or nefarious events was described among the unfavorable occurrences. Kodali et al. [102] achieved the same results by studying the high aortic rupture risk, coronary artery occlusion, and PVAR with the FEA method both in retrospective and prospective patients (*n* = 3). Of note, the simulation computational analysis revealed that the broad calcified aggregates placed inside the left coronary sinus between the coronary ostium and the aortic annulus were propelled by the stent, leading to aortic rupture. The most important consideration emerging from this study showed that the expected results from the simulations allowed a correct shared decision-making process once presented to the heart team clinicians. Therefore, engineering evaluation with FEA is recommended for rating patient-specific aortic rupture risk [102].

Robust evidence suggests that PVAR, rather than aortic rupture (aortic wall or annulus), as an emerged complication of TAVI, is associated with further worsening in late outcomes. The benefits of the application of the computational modeling of TAVI to high-risk patients, offering a quantitative evaluation of the area of perivalvular holes, become evident within the first post-operative 5-years, disclosing a survival advantage that tends to increase with time [9,10]. The location of incomplete adherence of the prosthetic stent to the aortic wall modifies the extent of the survival advantage of TAVI. Importantly, Morganti et al. suggested that the area of paravalvular holes was proportional to the volume of retrograde perivalvular blood flow and was in accordance with echocardiographic evidence [40,41].

Auricchio worked on measured eccentricity and stent configuration, revealing that the eccentricity of the deployed stent substantially affects valve closure and especially the coaptation of leaflets [103]. The evidence presented by Morganti et al. indicates that non-symmetric closure is attributed to elliptical stent configuration, leading to the incongruity that one leaflet can close under the other two. Again, although a small central gap may be generated, thus causing a regurgitant flow, the geometrical asymmetry of a stent is a crucial determinant of the central gap during diastole, and it is related to the choice of the leaflet material model. The latter has been shown to have a substantial impact on the coaptation values, being able to alter the early and long-term results [104,105]. 

Seven years after Auricchio et al., Bianchi et al. [42] evaluated post-procedural complications such as PVAR and related thromboembolic events that have been hampering the spread of the TAVI procedure in lower-risk patients receiving the last generation of the device. Finite element analysis and computational fluid dynamics analysis were performed in recipients of either Edwards SAPIEN or Medtronic CoreValve. The engineering-based simulation revealed that parametric analyses directly affected positioning and balloon over-expansion, thus suggesting a direct impact on the post-deployment TAVI performance to reach a maximum of 47% in the reduction in the PVAR volume [42].

Dowling et al. [49] used patient-specific computer simulations for TAVI in patients with clinically bicuspid aortic valve (BAV) morphology who were deemed suitable for the TAVI procedure and enrolled nine individuals in the study. Computational analysis simulation was effective for eight patients (89%) who required a change in treatment approach with self-expanded TAVR Evolut and Evolut PRO (Medtronic, Minneapolis, Minnesota). The evidence from simulations suggested the occurrence of moderate to PVAR for three recipients after the use of TAV, which were re-discussed by the heart team and considered for SAVR. As for the remaining six patients, the percutaneous treatment strategy was modified. Five patients who received TAVI (83%) with a self-expanding THV had altered size and/or implantation depth to minimize paravalvular regurgitation and/or conduction disturbance. In one patient, the computed analysis was performed, and significant conduction disturbance occurred after TAVI, requiring a permanent pacemaker that was inserted before mechanical intervention. Concerns about PVAR onset were correlated with no recurrence to the mild recurrence of AV regurgitation in all nine individuals. Note that the patient who required a pre-procedure permanent pacemaker implant with definitive dependent pacing revealed a conduction disturbance classified as a third-degree atrioventricular block. The investigators highlighted the remarkable value of the use of FEA simulation applied to TAVI in BAV which may predict important clinical outcomes, such as PVAR and conduction disturbance [49].

Finally, modern platforms to treat structural heart valve disease should entail the use of computational biomodelling, at least in the presence of major clinical or anatomic contraindications, and substantial efforts should be made to integrate computational biomodelling into MDCT and 3D echocardiography during TAVI procedures, avoiding the concern related to a central mild intraprosthetic leak [39,95,96,97,98,99,100]. Therefore, the scant evidence produced, which offers a comprehensive analysis of the effect of procedural parameters on patient-specific post-TAVR hemodynamics, limits the correct assessment of the effect of the TAV implant depth and balloon over-inflation on anchoring the stent. Ultimately, the occurrence of post-distribution PVL and the risk of thrombus formation remain the true Achilles’ heel. A deeper direct analysis of the aforementioned objectives can offer valid help to understand the effect of the choice of the interventional cardiologist on post-procedural complications and help reduce their impact on the basis of patient-specific data [40,41,42,43,50].

## 4. Discussion

### 4.1. Evidence to Deploy Biomechanical Evaluation and to Definitively Accept the Use of Transcatheter Heart Valve Implantation as a New Paradigm Shift

Both cardiology and cardiovascular surgery have witnessed an era of consistently evolving change, and this new scenario has mainly been driven by the emergence of percutaneous coronary intervention, with novel options for the treatment of coronary heart disease. The new endovascular platforms have evolved rapidly and established themselves as vital cogs in the armamentarium available to address structural heart disease [106]. In the past ten years, the innovation has initially been primarily invested in the management of aortic valve stenosis and subsequently the pathological mitral valve with the progressive affirmation of transcatheter valve therapy (TVT) [22,24,60]. From the first experimental study by Bonhoeffer, who pioneered the transcatheter pulmonary valve implant, [53] the use of TVT to treat aortic valve stenosis progressed rapidly. In 2010, the first PARTNER (Placement of AoRTic TraNs cathetER Valve Trial) reported a series of high-risk patients who were treated using this novel technique as opposed to conventional aortic valve stenosis surgery [3]. In less than 10 years, PARTNER III affirmed the safety and efficacy of the transcatheter aortic valve replacement in low-risk patients [16]. It is conceivable that future generations of transcatheter valves with the advancement of device technology will herald improvements in the hemodynamic profile, longevity, and durability alongside reduced adverse events.

Thomas Kuhn, an American physicist and philosopher, introduced the term “paradigm shift” for the first time in *The Structure of Scientific Revolutions* in 1962 [107]. In this report, the author explained how a process can lead to a transition from the previously widely accepted worldview to a new model for demonstrating new emerging evidence. Cardiology and cardiovascular surgery have often faced paradigm shifts because these disciplines are constantly open to a transition that has, over time, progressively fostered the innovative spirit of those who practice them. We can note that historically, numerous paradigm shifts emerged: coronary bypass grafting, heart transplantation, percutaneous coronary intervention, mechanical and bioprosthetic valves, generations of life-saving drugs for heart failure, and mechanical circulatory support [108,109]. The current summit of these advancements is the emergence of devices used for the replacement of the aortic valve with TVT. 

Calcific aortic valve stenosis (AVS) is a pathoanatomic process of aortic valve leaflets that are affected by structural changes sustained by an inflammatory and atherosclerotic process associated with calcium deposition. The morphological changes generated at the level of the cusps alter the function of the valve with a consequent reduction in the opening of the variably narrow leaflets during systole. Aortic valve disease causes abnormal hemodynamics and increased mechanical stress on the left ventricle (LV) [110]. 

Prior to the advent of TAVI, surgical aortic valve replacement (SAVR) was considered the ideal treatment option for patients at risk of severe valve obstruction. However, new platforms for the treatment of structural heart diseases have fueled clinical attention that has shifted towards the use of new less invasive armamentarium represented by THV devices.

The PARTNER Ia study proved the superiority of the transcatheter balloon-expanded procedure in patients receiving TAVI over those who were managed with optimal medical therapy in short- and medium-term mortality (43.3% in the TAVI group and 68.0% in the standard-therapy group (*p* < 0.001, at 2 years, respectively) [5]. As for prohibitive/high-risk patients with severe AVS who were suitable to receive surgical treatment, the use of TAVI revealed the same mortality at 5 years as compared to SAVR (67.8% TAVR cohort vs. 62.4% SAVR). However, patients who received TAVI disclosed a rate of moderate to severe AVR of 14% as compared to 1% in those receiving SAVR [9]. Not least, evidence from the use of a first-generation CoreValve Self-Expanding System revealed that the 1-year all-cause death rate was higher in patients after SAVR as compared to recipients of TAVI [8]. 

THVT has proven to be a revolutionary and decisive procedure in the last decade thanks to the achievement of efficacy and safety. In fact, evidence from THVT offered a clear answer to the use of the only life-saving solution for high- and extreme-surgical-risk patients who cannot tolerate the open surgical option due to the presence of significant comorbidities [111]. Given the promising results associated with technological advancement which has undergone very rapid development, the use of TAVI has been approved for the treatment of intermediate-risk patients. The results reported by the pioneering RCTs suggested increased rates of residual aortic valve regurgitation and more pacemakers implanted in the population intended for the TAVI procedure; however, the use of THVT was directed toward the design of randomized trials involving the intermediate/low-surgical-risk population [9,10,13,15,16,17]. 

The SURTAVI trial enrolled 1660 patients who were eligible to receive either transcatheter aortic-valve bioprosthesis (*n* = 864) or SAVR with the standard procedure (*n* = 796). All patients were symptomatic of severe aortic stenosis at intermediate surgical risk. The primary objective was to demonstrate the non-inferiority, safety, and efficacy of the first and second generations of the CoreValve System [15].

In SURTAVI, 84% of patients were managed with the first-generation CoreValve System while 16% of recipients of TAVI had the second generation of Evolut R bioprosthesis. This cohort of individuals had an STS score Society for Predicted Risk of Mortality at 4.5 ± 1.6% [15].

At 2 years, the results revealed that the composite of death from any cause or disabling stroke was higher in the SAVR group as compared to the TAVI group (14% vs. 12.6%, respectively) [15]. The New York Heart Association values for clinical symptoms were significantly improved in both cohorts compared to pre-operative data and were consistent throughout the 24-month follow-up. In addition, the KCCQ summary score revealed a substantial and stable improvement in both populations at 2 years of follow-up, although patients managed with the TAVI procedure had a greater percentage of improvement at 1 month than those who received a standard aortic valve replacement [15].

Evidence of the non-inferiority of TAVI over SAVR recorded for intermediate and high-risk patients offered favorable points to undertake the randomized PARTNER 3 trial [16] and the multi-national randomized clinical Evolut Low Risk Trial Investigators 26 for patients presenting with severe AVS at low risk for death after surgical procedure [17]. In the third series of results reported from the two RCTs, the composite of death from any cause, stroke, or re-hospitalization at 1 year was less in TAVI recipients after the implantation of the device. Again, the investigators found shorter hospitalization rates for individuals undergoing TAVI, while there were no significant differences between groups in terms of major vascular complications, new permanent pacemaker insertions, or moderate or severe paravalvular regurgitation [16,17].

Certainly, a decisive impetus for the success of the large-scale TVT procedure has been linked to refined technological progress, which has seen the use of introducers of reduced diameter and an improvement in the use of stents which have proved to be safer and more effective. However, it is important to consider that the results must be confirmed by longer-term follow-ups.

### 4.2. Biomechanics Computational Modeling to Give Consistency to The Paradigm Shift 

#### 4.2.1. Paravalvular Aortic Regurgitation

Although there has been substantial initial growth in the use of TAVI confirmed by the success of the results, intra- and post-procedural clinical complications have questioned the paradigm shift, questioning the potential expansion of TVT in low-risk patients. 

We have learned that post-deployment PVAR, cardiac conduction abnormalities [112,113] (Bagur et al., 2012; Van der Boon et al., 2012), and coronary artery occlusion (Ribeiro) are among the most marked immediately recorded disadvantages [114]. Taken together, these complications revealed an increased rate of mortality and reoperation [23,112,113,114].

Surely the Achilles’ heel of the TAVI is constituted by the altered hemodynamics due to the occurrence of PVAR, in which the emergence of narrow gaps which are exposed to high gradients of systolic pressure can lead to an altered function of the platelets, which are therefore exposed to high flow shear stress. This pathoanatomic condition triggers platelet activation, perturbing the aggregation/coagulation balance, with the formation of microemboli. The latter are then expelled at the next systole and can remain trapped and/or deposited in the region of the Valsalva sinuses, which offer a suitable location for typical low-shear recirculation areas. Therefore, PVAR may be linked to the deposition of thrombi around the THV device as well as to the potential circulation of thromboembolic clots, which is followed by an increased risk of stroke. Several pieces of evidence have reported that thromboembolism is less common than the hypo-attenuated thickening of the leaflets; however, it is still a fairly common and dangerous phenomenon that requires adequate clinical treatment [115]. Another point to consider is the close association of leaflet thrombosis and the development of a structural degeneration of the valve incorporated in the device.

Several studies have suggested that the occurrence of PVAR in recipients of the TAVI procedure is directly correlated with higher late mortality, cardiac death, and repeated hospitalization even in the presence of traces of regurgitation [116]. Five-year results from Partner Ib RCT disclosed a rate of 14% moderate or severe aortic regurgitation in patients who received TAVI as compared to those who were managed with SAVR. This evidence caused an increased risk of mortality at 5 years for patients who developed moderate or severe aortic regurgitation after TAVI [9].

All the indicators testify that the mortality rate was proportional to the severity of the regurgitation, and in this regard, Generaux et al. [35] reported that even slight PVAR can lead to a doubling of the mortality rate after 1 year. However, Webb et al. [2] pointed out that the progression of PVAR can be unpredictable. The investigators observed that at 2 years, regurgitation increased by ≥ 1 grade in 22.4% of patients, remained unchanged in 46.2%, and improved by ≥ 1 grade in 31.5%.

In this context, substantial differences emerged after the installation of a balloon-expandable THV device or the use of the self-expandable valve. Two independent studies revealed that recipients of the Medtronic CoreValve self-expanding device experienced a higher PVL rate and worsening severity than patients who received an expandable Edwards SAPIEN balloon [50,117]. However, substantial improvements have been made in the new devices involving the low-profile delivery system and external skirt, thereby improving the sealing of the THV device and promoting more precise valve positioning. A lower rate of PVAR at short-term follow-up has been reported [118].

Patients who exhibit PVAR post-TAVI require clinical and imaging modality evaluation. The quantification of regurgitation is generally determined with the use of echocardiography.

In detail, methods such as transesophageal echocardiography, cineangiography, and hemodynamic measurements are commonly used during the procedure, while transthoracic echocardiography offers substantial support for the evaluation and follow-up of PVAR after TAVI [119]. Above all, the continuous wave echo is the most commonly used method to evaluate the overall hemodynamic performance of the valve, but with the disadvantage of not being able to obtain a spatial localization of leaks. The relative consequence is that aortic regurgitation is quantified as the ratio of reverse flow to direct flow. As reported by Hatoum et al. [120], the most obvious limitation is that the measurement and determination are experimental. However, a semi-quantitative description of jets by pulsed wave color Doppler can be used to obtain a precise localization and evaluation of the gravity of PVAR jets.

Concern related to the quantification of PVAR persists after TAVI due to a lack of standardization, leading to a challenging diagnosis. In fact, it is often qualitative, and different classification schemes are adopted (trace, mild, moderate, and severe) [119,121]. Several interventional alternatives to reduce paravalvular regurgitation have been put in place and include post-implantation balloon dilation, repositioning, entrapment maneuvers as well as the valve-in-valve (ViV) procedure [122]; all of these are not free from an increasing risk of vascular complications. A critical aspect of the procedure is represented by the positioning of the THV device with respect to the patient’s aortic annulus, which was directly associated with the degree of hemodynamic performance of TAVI as well as the rate of reintervention [123]. There is early evidence from Nombela Franco et al. [124] and Takagi et al. [125] who reported that balloon over-inflation is often used to reduce the degree of PVAR. The investigators revealed the post-balloon dilation decreases regurgitation in the preponderance of patients by at least one degree [124,125]. However, how crucial the post-dilation effect is on survival remains elusive. Again, an association with a higher incidence of cerebrovascular events was recorded [124]. The goal of a correctly performed transcatheter procedure necessarily involves minimizing the amount and incidence of PVAR in order to gain improved clinical outcomes in the long term.

The development of computational models was identified early as the correct method of studying the interaction between TAVI stents and native aortic tissue and predict the performance of the post-procedural device from the point of view of structural dynamics [41,43,47,126,127]. Recently, several studies have substantially quantified the degree of interaction between the device and the implantation site, as a surrogate measure of PVAR, by measuring the gap between the stent [40,48] or the skirt [128] from native tissue, considering the specific anatomical characteristics of the patient’s aortic root. Chang et al. reported ideal characteristics that offer better results in terms of PVAR occurrence [129]. We compared the two most commonly used devices, documenting a better performance of the third generation of the balloon-expandable device compared to the third generation of the self-expandable device in adapting to the dynamics of the aortic root, reducing the risk of PVAR [50].

Similarly, great interest has been aroused in the creation of a maximum flow algorithm [46], producing a one-dimensional connected graph capable of representing the flow network based on the size of the gap existing between the stent and the aortic root. Although in the absence of PVAR the results showed a good correlation, nevertheless, the reliability was reduced with the development of models that lacked precision for patients with PVAR recurrence. A significant report was described by De Jaegere et al. [44], who referred to a large series of computational models that tested the predictability of 60 Medtronic CoreValve deployment cases in which the results were validated through angiographic and echocardiographic measurements. The limitation of the work lay in the lack of an adequate description of the reconstruction of the patient’s anatomy with respect to the modeling hypotheses. Finally, in a recent study, Mao et al. [130] evaluated the effect of CoreValve orientation and modeling assumptions, such as skirt shape and stent thickness, on post-deployment hemodynamics. However, the formation of post-TAVI thrombus only involved the generated clots on the valve leaflets following a ViV procedure. Vahidkhah et al. analyzed blood stasis by assessing and quantifying idealized ViV models with intra-annular and supra-annular TAVI positions [131].

#### 4.2.2. Transcatheter Heart Valve Thrombosis

Evidence based on several reports displayed that recipients of TAVI experienced an unclear rate of bioprosthetic valve thrombosis (BPV-TH) and thromboembolic complications of the device. It is of note that both results from the RCTs and EU Partner Registry lack complete and satisfactory data. The PARTNER and CoreValve System randomized clinical trials did not note significant BPV-TH [9,10,25]. On the other hand, the EU Partner Registry [132] also revealed very poor data on thromboembolic events in patients who were managed with THV devices. The reported thromboembolic complication rate was only 1 case out of 130 patients undergoing TAVI. Latib et al. noted that from a large number of patients (*n* = 4266), only 27 cases of BPV-TH thrombosis (0.61%) occurred within a median of 181 days after TAVI procedure [132]. 

Importantly, Stortecky et al. observed that the risk of BPV-TH was higher in the first 3 months after device implantation. In addition, the risk curves showed a marked reduction in events in the subsequent months, which almost matched the curves of the general population [133]. A histopathological analysis from the CoreValve device thrombotic complication suggested that clot formation was completed approximately 3 months after the implantation of the THV device [134,135,136,137,138]. Makkar et al. [139] offered important data systematically using 4D computed tomography to prove bioprosthetic valve thrombosis events. Fifty-five patients included in the PORTICO Studio IDE (Portico Re-sheathable Transcatheter Aortic Valve System US IDE Trial) revealed the occurrence of BPV-TH at a median of 32 days after valve implantation with decreased leaflets movement in 40% of recipients. In total, 132 patients were included in the Savory study (subclinical aortic valve thrombosis assessed with 4D CT) and were eligible to receive either TAVI or SAVR, or were included in RESOLVE (surgical catheter and aortic evaluation of thrombosis of the bioprosthetic valve and its treatment with anticoagulation) and underwent 4D computed tomography within 3 months, recording reduced leaflet motion at a rate of 13% of recipients. Of these, 14% were treated with TVI, while 7% underwent SAVR with the use of a conventional bioprosthesis [139,140].

Pache et al. [141] corroborated the previous evidence [139,142] on 156 consecutive patients who were managed with TAVI using SAPIEN 3 (Edwards Lifesciences, Irvine, CA, USA). At a median of 5 days after the procedure, the investigators observed by the mean of multi-detector computed tomography that 10.3% of TAVI recipients disclosed leaflet thickening with hypo-attenuation. Although the absence of symptoms was considered a relevant point for a normal clinical evolution, individuals experienced a higher mean transvalvular gradient, and anticoagulant drug therapy led to the complete resolution of leaflet thickening [141]. Likewise, in patients who were treated with dual antiplatelet therapy (DAPT) less frequently than those who were managed with a single antiplatelet drug (37.5% and 50%, respectively) [141], a correlation between increased transvalvular gradient and uncontrolled neointimal proliferation was noted with thickening of the device leaflets [141,142].

Three recent studies reached significant relevance in BPV-TH and thromboembolic events [135,143,144]. Hansson et al. [135] monitored patients who underwent a TAVI procedure with the use of balloon-expandable valves (Edwards Sapien XT or Sapien 3 valves) by means of transthoracic or transesophageal echocardiography and multi-detector computed tomography to screen the incidence and predictors of BPV-TH at 1–3 months. The evidence of thrombosis was observed in a rate of 7% of patients with MDCT. In addition, 18% of individuals experienced bioprosthetic valve thrombosis events with clinical complications. Cox’s multi-variate regression analysis revealed that the two independent predictors of BPV-TH were related to the use of the TAVI and were the identified in the lack of warfarin administration and the larger size of the device measured at 29 mm [135].

Nührenberg et al. [143] studied hypo-attenuated leaflet thickening (HLAT) as a potential precursor of clot formation and thromboembolic events after TAVI. In all cohorts of patients, including those who underwent oral anticoagulation treatment, dual antiplatelet therapy with aspirin and clopidogrel was administered for at least 24 h before the procedure. In patients who had pre-existing indications for oral anticoagulation treatment, aspirin was discontinued, and the administration was pursued after TAVI for the rest of the cohort. Additionally, 18% of TAVI patients revealed hypo-attenuated leaflet thickening; however, lower complication rates were observed in patients receiving oral anticoagulation, suggesting that the administration of dual antiplatelet therapy (aspirin and clopidogrel) did not change the occurrence of early HLAT [143].

GALILEO 4D RCT [144] included 231 patients for antithrombotic strategy assessment, in which long-term anticoagulation was administered, either with the use of rivaroxaban (10 mg) associated with aspirin (75 to 100 mg) once daily or with the administration of a dual antiplatelet-based strategy with the use of (clopidogrel (75 mg) plus aspirin (75 to 100 mg) once daily. Four-dimensional CT was used after randomization to check all cohorts of individuals. Patients were successfully treated with TAVI with no indication for long-term anticoagulation therapy. The primary endpoint of the study comprehended the percentage of patients who experienced at least one prosthetic valve leaflet with grade 3 or higher motion reduction. Of note, this process involved substantially more than 50% of the leaflet as follows: 2.1% of patients with rivaroxaban administration revealed at least one prosthetic valve leaflet with grade 3 or higher motion reduction compared to 10.9% in the dual antiplatelet protocol. The thickening of at least one leaflet was recorded in 12.4% of patients in the rivaroxaban cohort compared to 32.4% in which the dual antiplatelet was administered. Lastly, concerns about the increased risk of death or thromboembolic events and the risk of life-threatening or disabling events, or greater bleeding were remarkably higher in patients who received the rivaroxaban administration [144].

One of the concerns affecting clot formation after the TAVI procedure is related both to the extent of bulky native valve calcification and its position with respect to the annulus of AV and the aortic root, as well as to stent deformation and the size of the patient’s annulus. Even more so, in these specific morphological features, the role of physiological blood dynamics plays a crucial role that has not been fully investigated [39].

Khalique et al. [145] noted that calcified blocks substantially affect the amount and asymmetry depending on the extent of aortic valve calcification. This condition led to the involvement of all regions of the aortic valve complex in predicting various grades of PVAR from greater than or equal to mild PAVR and the post-deployment performance of the device, thereby potentially evolving towards the bioprosthetic valve thrombosis of the THV device. The preexistent leaflet asymmetry was excluded so as to confirm the diagnosis of PAVR. The quantity of bulky calcification at the level of the junction between the annulus and LVOT, as well as the occurrence of leaflet calcification, independently predicted PVAR and the post-deployment of TAVI when taking into account the multi-detector row computed tomography area cover index [145].

For this reason, the use of computational biomodelling can lead to predicting both the extent of PVAR and the risk of clot formation [39,40,41,42,50,51,52]. Likewise, the bulky calcification penetrating the aortic annulus may have a different texture, thus raising some reflections about the ideal choice of device to implant [40,41,50,145]. So, the use of self- and balloon-expandable system prostheses can lead to different geometric alterations of the aortic annulus after deployment, with a greater or lesser risk of potential disturbance of the blood fluid dynamics that generate clot formation [5,40,41,42].

**Figure 8:**
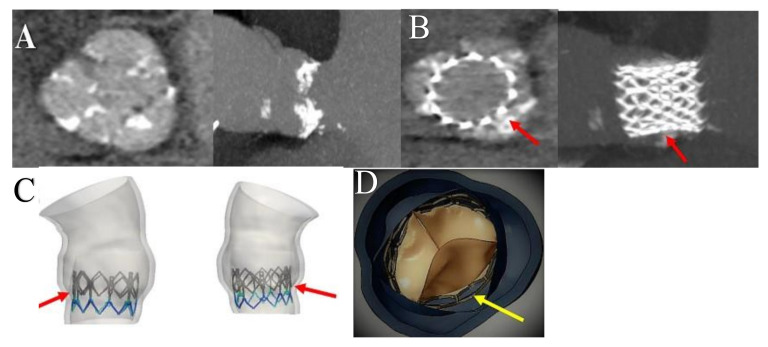
Depict preoperative (**A**) and postoperative (**B**) 3 D CT scan with TAVI thrombosis. (**C**) Biomodelling of a Sapien XT reveals an incomplete deployment (red arrow) of the device with PAVR and thrombotic formation (red arrow). (**D**) The yellow arrow disclose a distortion of the stent and reduced mobility of the leaflet of the bioprosthesis in correspondence of the PAVR. Abbreviations; PAVR paravalvular aortic regurgitation. Other abbreviations in previous figures.

In this regard, we revealed that both balloon- and self-expandable devices were poorly effective in the presence of bulky native AV calcifications, and the different degrees of device deformation were studied. Two independent reports based on computational biomodelling suggested that both Sapien XT and Sapien 3 disclosed high values of the maximal principal stress in the aortic regions close to bulky calcification, resulting in a deformation of the stent that assumed an elliptical shape [40,52]. Accentuated geometric modification with incorrect post-deployment can lead to paravalvular leakage, leaflet mal-coaptation, and hypo-attenuated leaflet thickening. The extreme shape of elliptical deformation is likely to favor subclinical thrombosis due to the presence of residual calcifications that favor hypomobility [40,52]. The SAPIEN device is shown in Figure 8. 

Again, the core valve is based on the self-expansion mechanism that may succumb to the mechanical distortion phenomena. In self-expanding TAVI, the crucial role of positioning in determining valve anchorage is pivotal. The occurrence of non-uniform expansion related to extensive calcifications can lead to prosthetic device deformation that ranges from an increased eccentricity > 10%, resulting in the incomplete expansion of the nitinol frame at almost all levels and potentially causing clot formation [41,42,50].

No evidence has demonstrated a statistically significant correlation between the occurrence of moderate PVAR and abnormal flow patterns on the TAV implanted leaflets and in the left main coronary artery that could favor thrombosis of the THV device and the accelerated progression of the atherosclerotic process [146]. However, several observations suggest that clot formation has been hypothesized to be more directly related to PVAR with the clinical occurrence of a thrombotic embolism [52,135,139,140,141,142,143,144].

An explanation can be offered by the existence of localized flow at the PVAR level with the development of high-pressure gradients associated with the presence of small, tight, empty areas. This condition implies that the platelets are subjected to high flow shear stress [41,42,52]. This phenomenon, as we have reported, has attracted ever-increasing clinical interest [41,52].

Bianchi et al. [42] evaluated the relationship between PVAR and platelet activation with a computational model to study the thrombogenic potential of three procedural configurations of TAVI, two of which were Sapien 3 and one was CoreValve Evolute. Investigators calculated the stress accumulation of platelets along particle trajectories in the PVAR region. All the probability density functions in the three simulations performed showed comparable patterns. For example, in one Sapien 3 with a valve measured 26 mm, in which an over-inflated aortic configuration was exhibited, the major stress accumulation of platelets was evident. This phenomenon can be related to the higher speed that can be recorded in PVAR jets, which leads to higher flow shear stress. In addition, HS values were observed to be in agreement with the largest overall regurgitation volumes. The information obtained from the probability density functions showed that the variation in the diameter of PVAR affects the activation potential of platelets. For example, in CoreValve Evolut 29, a reduction in PVAR grade led to slightly higher thrombogenic potential, as platelets were subjected to more shear stress which was related to their flow through smaller paravalvular spaces [42]. Finally, dynamic fluid has also shown us that when the volume of regurgitation is considerably higher, the cause–effect relationship established between PVAR reduction and susceptibility to platelet activation is supported by a more complicated interaction [41,42,52].

#### 4.2.3. Structural Valve Degeneration

The term structural valve degeneration (SVD) implies an acquired anomaly of the valve bioprosthesis due to a substantial deterioration of the flaps and of the structural support that integrates the device. The correlated patho-anatomic consequence is the thickening, calcification, laceration, or rupture of the materials that make up the valve prosthesis. This context of the pathological disorder suggests the development of associated valvular hemodynamic dysfunction, such as the development of stenosis or regurgitation. To date, a thorough understanding of the precise mechanisms underlying SVD has not yet been substantially offered. However, the mechanisms that support SVD are multiple, both mechanical and related to fluid dynamics, which are responsible for tissue rupture or thickening over time [27,28,29,30,31,32,33,147,148,149,150,151,152,153,154,155,156,157,158,159,160,161,162,163,164,165,166,167,168,169,170].

Several factors cause SVD. First of all, a crucial role is provided by the mechanical stress levels associated with both flow anomalies and the occurrence of shear stresses on the surfaces of valve leaflets. These two factors are potentially responsible for the progression of SVD, leading to the breakdown of the collagen frame of the fibers and the calcification of the tissues [159,171]. Second, other clinical conditions, in which the pathological features of intrinsic structural deterioration of the valve tissue are not detectable, cannot be classified as SVD. However, they deserve to be taken into consideration. SVD may be related to the mismatch between prosthesis size and patient size, device malposition, paravalvular regurgitation, and abnormal frame expansion. Likewise, these abnormal situations attributable to the implanted bioprosthesis can lead to early SVD or be considered a cause of its development. Dysfunction involving the prosthesis implanted due to mismatch is difficult to distinguish from the structural degeneration of a valve. Therefore, it is not considered to be SVD as it exhibits normal leaflet morphology, but instead has a valve area that is relatively small with a high gradient [27,28,29,30,31,32,33,147,148,149,150,151,152,153,154,155,156,157,158,159,160,161,162,163,164,165,166,167,168,169,170].

**Figure 9:**
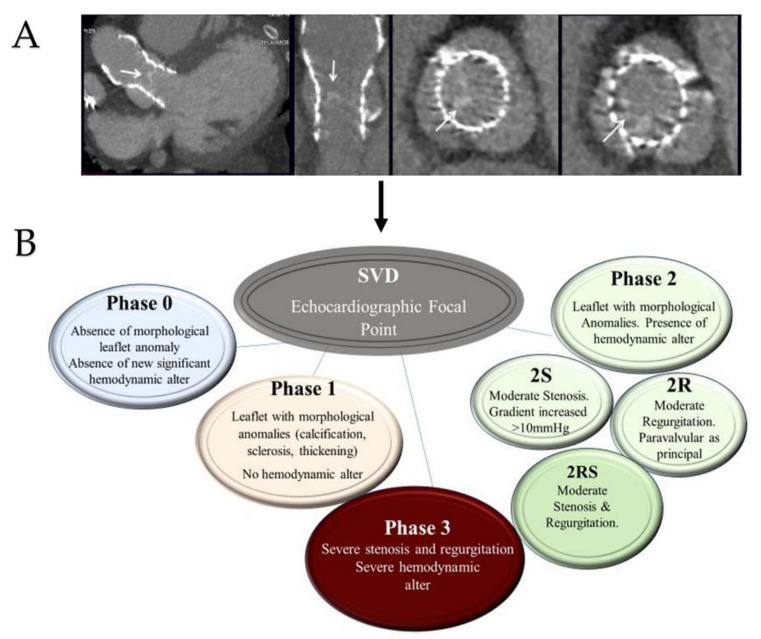
(**A**): Early SVD with calcifications (white arrow) of TAVI in patient receiving self-expanded first-generation CoreValve 26 mm (CoreValve, Minneapolis, Minnesota). (**B**): Classification of SVR based on recommendations of the VARC-2 for stent/stentless xenograft. The useful elements to define SVD as valve-related dysfunction were the mean aortic gradient ≥20 mm Hg, the effective orifice area ≤0.9–1.1 cm^2^, a dimensionless valve index <0.35 m/s, and moderate or severe prosthetic regurgitation. Phase 0 displays the absence of morphological leaflet anomaly and absence of hemodynamic alteration. Phase 1 discloses early morphological changes without hemodynamic compromise. The morphological alterations typical of stage 1 are also referable to prostheses where the degenerative process is controlled using antithrombotic drugs that reduce the thickening of the leaflet. Phase 2 reveals morphological abnormalities of valve leaflets of SVD associated with hemodynamic dysfunction. The bioprosthesis in this phase can manifest as stenosis or regurgitation. The thrombosis is a factor favoring phase 2, leading to stenosis or paravalvular leakage and regurgitation. Phase 2 includes two subcategories, phase 2S and phase 2R. In the evolutive stage of 2S degeneration, an increase in the mean transvalvular gradient (≥10 mm Hg) and decrease in the valvular area without leaflet thickening occur. SVD may occur in the 2RS form including moderate stenosis and moderate regurgitation. Phase 3 of SVD highlights severe stenosis or severe regurgitation with severe hemodynamic change. Abbreviations: R, regurgitation; SVR, structural valve degeneration; S, stenosis; VARC, Valve Academy Research Consortium.

A crucial point that characterizes the difference between the prosthetic mismatch and the SVD is related to the time during which the anomaly is established. The prosthetic maladjustment reveals hemodynamic anomalies of the valve which occur at the moment of the implantation of the prosthesis with the manifestation of the patient’s hemodynamic deterioration, which occurs in conjunction with an increase in gradients and a decrease in the valve area; these conditions reveal a progressive increase in the patient’s clinical conditions on repeated echocardiographic checks. In patients who develop SVD, associated stenosis develops progressively and is seen with the characteristics of a faded lesion during follow-up. Although both prosthetic valve thrombosis and infective endocarditis are not included in the definition of SVD, SVD may be noted despite having recorded therapeutic success. Intense debate currently surrounds SVD due to its potential to involve and therefore influence the TAVI procedure [147,148,149,150,151,152,153,154,155,156,157,158,159,160,161,162,163,164,165,166,167,168,169,170]. Indeed, since a less invasive transcatheter approach is available for patients presenting with comorbidities and at high risk with conventional surgical strategies, fewer cases of SVD were detected, possibly because the deceased patients were not included in the long-term follow-up. Cardiologists believe that SVD is not a reliable criterion for establishing true biological valve durability. They suggested that the actuarial freedom found by re-intervention is inherently lower than the freedom from SVD [147,148] (Figure 9).

Only the NOTION RCT [31] with 6 years of follow-up disclosed SVD rates that were significantly greater after SAVR than the TAVI procedure (24.0% vs. 4.8%; *p* < 0.001). The investigators reported in post-procedural echocardiographic controls a mean gradient of >20 mm Hg in 22% of patients who experienced SVD compared to 2.9% for those who were managed with TAVI (*p* < 0.0001). This evidence was also corroborated at a 3-month post-procedure check where a modified definition of SVD was fixed and a mean gradient increase >10 mmHg was established (AVR-S 12.4% vs. TAVR 1.4%; *p* < 0.001) [31].

In Figure 9 panel A an echocardiographic focal point of the SVD of the stent/stentless xenograft is depicted. 

On the other end, patients who were checked at a 5-year follow-up in the PARTNER trial disclosed no structural valve deterioration with the preservation of low gradients and increased valve areas [9,10]. The results of the two randomized studies are encouraging, but a longer follow-up is necessary to confirm and give more solidity in terms of the safety and effectiveness of the transcatheter procedure [9,10].

The bioprosthesis designed as part of the Sapien THV balloon-expandable device consists of bovine pericardium as opposed to calf pericardium which characterizes the surgically implanted Edwards bioprosthesis. However, it should be noted that the treatment procedure is identical [171]. The use of the TAVR 22 Fr and 24 Fr systems has been adapted to the leaflets of the TAV, which are thinner than surgical bioprosthesis. Rapid technological advances have led to the development of delivery systems reduced to 18 Fr before and 4 Fr after for the second generation of Sapien XT and for the third-generation Sapien 3 (Edwards Lifesciences, Inc.), which accompanied the changes made to the stent in cobalt–chromium and thinner leaflets to obtain a lower crimped TAV profile.

The study by Xuan et al. [56] revealed that the major and minor stresses in the Sapien 26 mm valves are located proximally in the annulus, where the stent is deployed and narrowed. The investigators highlighted that maximum and minimum principal stresses were exhibited at the level of TAV leaflets that were attached to the stent located in close contact with the commissures. It is reasonable to suggest that these regions where the peak stress and the highest stress levels occur locally could result in the areas most prone to initiate degeneration. To date, we have no knowledge of studies that have shared a comparison on the relative duration of TAVI compared to surgical bioprosthesis. Evidence reported from studies on the degeneration of surgical bioprosthesis suggests that degeneration associated with calcification or tearing of the flaps correlates with areas of high tensile and compressive stresses [56].

Sun et al. [172] performed the first computational biomodelling using FEA on two bovine pericardial valves from Edwards Lifesciences Inc. The test was performed with quasi-static loading conditions set below 120 mm Hg, with leaflet material properties fixed from those valves and respecting the exact valve geometry 11. The investigators recorded a maximum in the plane stress that ranged from 544.7 kilopascals (kPa) to 663.2 kPa, reliant on the material properties of the leaflet were used. Of note, the degree of stress had different locations. In fact, they revealed that the stresses on the leaflets were greatest near the commissures and inferior near the free edge of the leaflet. In a subsequent study, the authors reported the results of an FEA simulation performed on a 25 mm surgical bioprosthesis, which is the closest dimension to the size of the commonly implanted Sapien balloon-expandable device. Again, Xuan et al. [56] suggested levels of maximum principal stress for a 26 mm Sapien valve that were significantly higher than those recorded for a surgical bioprosthesis, offering an explanation due to the difference in the design of the leaflets or different interaction with the respective frame that constitutes the device [56]. Alavi et al. revealed that the crimping process physically damages TAV leaflets and may undermine leaflets, leading to increased leaflet stress [173].

## 5. Conclusions 

TAVI and SAVR are both options that should be seen as part of the treatment armamentarium offered to patients. Future research should be focused on detecting and addressing cumbersome calcium deposits which may increase the risk of paravalvular leaks, early valve degeneration, and permanent pacemaker insertion The use of adjuncts such as FEA and MDCT can help steer the decision-making process of heart teams while considering the patients’ wishes. Although currently comparable, the long-term effects of TAVI are still uncertain, but advancements are being made at a rapid rate to ensure it remains a pivotal option for treating aortic valve stenosis. Further longitudinal studies are also needed to assess the long-term outcomes of TAVI valves vs. SAVR. 

## 6. Limitations 

There are several limitations to this review in that it is by no means a systematic review or metanalysis. The heterogeneity of the studies paralleled with the advancement of valves makes direct comparisons unreliable. To ensure the material presented was up to date, only recently published papers were used with the addition of well-cited older articles. The use of finite element analysis is also limited in the clinical setting, with few centers offering this. Studies assessing the impact of TAVI on the other valves during implantation are also scarce. Given the recent emergence of TAVI, direct comparisons to SAVR may be limited by intangibles such as increasingly diligent follow-ups compared to routine standard of care. 

## Abbreviations

ACC	American College of Cardiology
AHA	American Heart Association
AR	aortic regurgitation
AVS	aortic valve stenosis
CT	computed tomography
COR	class of recommendation
DLP	Dose Length Product
ESC	European Society of Cardiology
FEA	finite element analysis
HF	heart failure
LOE	level of evidence
LVF	left ventricular function
mGy	microgray
MDCT	multi-detector row computed tomography
PA	pulmonary artery
PAVR	paravalvular aortic regurgitation
RCT	randomized clinical trial
STL	stereolitographic
SVD	structural valve degeneration
SAVR	surgical aortic valve replacement
TAVI	transcatheter aortic valve implantation
TVT	transcatheter valve therapy
THV	transcatheter heart valve
VARC	Valve Academic Research Consortium

## References

[1] Cribier A., Eltchaninoff H., Bash A., Borenstein N., Tron C., Bauer F. (2002). Percutaneous transcatheter implantation of an aortic valve prosthesis for calcific aortic stenosis first human case description. Circulation.

[2] Webb J.G., Altwegg L., Boone R.H. (2009). Transcatheter aortic valve implantation: Impact on clinical and valve-related outcomes. Circulation.

[3] Leon M.B., Smith C.R., Mack M., Miller D.C., PARTNER Trial Investigators (2010). Transcatheter aortic-valve implantation for aortic stenosis in patients who cannot undergo surgery. N. Engl. J. Med..

[4] Smith C.R., Leon M.B., Mack M.J., PARTNER Trial Investigators (2011). Transcatheter versus surgical aortic-valve replacement in high-risk Patients. N. Engl. J. Med..

[5] Makkar R.R., Fontana G.P., Jilaihawi H. (2012). Transcatheter aortic-valve replacement for inoperable severe aortic stenosis. N. Engl. J. Med..

[6] Kodali S.K., Williams M.R., Smith C.R. (2012). Two-year outcomes after transcatheter or surgical aortic-valve replacement. N. Engl. J. Med..

[7] Falk V. (2014). Transcatheter aortic valve replacement indications should not be expanded to lower-risk and younger patients. Circulation.

[8] Adams D.H., Popma J.J., Reardon M.J. (2014). Transcatheter aortic-valve replacement with a selfexpanding prosthesis. N. Engl. J. Med..

[9] Mack M.J., Leon M.B., Smith C.R. (2015). 5-year outcomes of transcatheter aortic valve replacement or surgical aortic valve replacement for high surgical risk patients with aortic stenosis (PARTNER 1), a randomised controlled trial. Lancet.

[10] Kapadia S.R., Leon M.B., Makkar R.R., PARTNER trial investigators (2015). 5-Year outcomes of transcatheter aortic valve replacement compared with standard treatment for patients with inoperable aortic stenosis (PARTNER 1), a randomised controlled trial. Lancet.

[11] Deeb G.M., Reardon M.J., Chetcuti S. (2016). 3-Year outcomes in high-risk patients who underwent surgical or transcatheter aortic valve replacement. J. Am. Coll. Cardiol..

[12] Siemieniuk R.A., Agoritsas T., Manja V. (2016). Transcatheter versus surgical aortic valve replacement in patients with severe aortic stenosis at low and intermediate risk: Systematic review and meta-analysis. BMJ.

[13] Leon M.B., Smith C.R., Mack M.J. (2016). Transcatheter or surgical aortic-valve replacement in intermediate risk patients. N. Engl. J. Med..

[14] Thourani V.H., Kodali S., Makkar R.R. (2016). Transcatheter aortic valve replacement versus surgical valve replacement in intermediate-risk patients: A propensity score analysis. Lancet.

[15] Reardon M.J., Van Mieghem N.M., Popma J.J., SURTAVI Investigators (2017). Surgical or Transcatheter Aortic-Valve Replacement in Intermediate-Risk Patients. N. Engl. J. Med..

[16] Mack M.J., Leon M.B., Thourani V.H. (2019). Transcatheter aortic-valve replacement with a balloon-expandable valve in low-risk patients. N. Engl. J. Med..

[17] Popma J.J., Deeb G.M., Yakubov S.J. (2019). Transcatheter aortic-valve replacement with a selfexpanding valve in low-risk patients. N. Engl. J. Med..

[18] Makkar R.R., Thourani V.H., Mack M.J., PARTNER 2 Investigators (2020). Five-Year Outcomes of Transcatheter or Surgical Aortic-Valve Replacement. N. Engl. J. Med..

[19] Otto C.M., Nishimura R.A., Bonow R.O., Carabello B.A., Erwin J.P., Gentile F. (2021). 2020 ACC/AHA guideline for the management of patients with valvular heart disease: Executive summary: A report of the American College of Cardiology/American Heart Association Joint Committee on clinical practice guidelines. J. Am. Coll. Cardiol..

[20] Vahanian A., Beyersdorf F., Praz F. (2022). 2021 ESC/EACTS Guidelines for the management of valvular heart disease. ESC/EACTS Scientific Document Group; ESC National Cardiac Societies. Eur. Heart J..

[21] Spadaccio C., Fraldi M., Sablayrolles J.L., Nappi F.J. (2016). TAVI in Lower Risk Patients: Revolution or Nonsense? Keep Calm and Select Patients. J. Am. Coll. Cardiol..

[22] Nappi F., Spadaccio C., Sablayrolles J.L. (2016). Pushing the Limits in Transcatheter Aortic Valve Replacement: High-Volume Center’s Effect, Overconfidence, or Something Else?. JACC Cardiovasc. Interv..

[23] Nappi F., Spadaccio C., Sablayrolles J.L. (2017). Delayed prosthesis malposition after transcatheter aortic valve implantation causing coronaries obstruction. Eur. J. Cardiothorac. Surg..

[24] Attias D., Nejjari M., Nappi F. (2018). How to treat severe symptomatic structural valve deterioration of aortic surgical bioprosthesis: Transcatheter valve-in-valve implantation or redo valve surgery?. Eur J Cardiothorac Surg..

[25] Reardon M.J., Adams D.H., Kleiman N.S. (2015). 2-year outcomes in patients undergoing surgical or selfexpanding transcatheter aortic valve replacement. J. Am. Coll. Cardiol..

[26] Siontis G.C.M., Overtchouk P., Cahill T.J. (2019). Transcatheter aortic valve implantation vs. Surgical aortic valve replacement for treatment of symptomatic severe aortic stenosis: An updated meta-analysis. Eur. Heart J..

[27] Didier R., Eltchaninoff H., Donzeau-Gouge P. (2018). Five-Year Clinical Outcome and Valve Durability After Transcatheter Aortic Valve Replacement in High-Risk Patients. Circulation.

[28] Panico R.A., Giannini C., De Carlo M. (2019). Long-term results and durability of the CoreValve transcatheter aortic bioprosthesis: Outcomes beyond five years. EuroIntervention.

[29] Durand E., Sokoloff A., Urena-Alcazar M. (2019). Assessment of Long-Term Structural Deterioration of Transcatheter Aortic Bioprosthetic Valves Using the New European Definition. Circ. Cardiovasc. Interv..

[30] Mack M., Carroll J.D., Thourani V., Vemulapalli S., Squiers J., Manandhar P. (2021). Transcatheter Mitral Valve Therapy in the United States: A Report From the STS-ACC TVT Registry. J. Am. Coll. Cardiol..

[31] Thyregod H.G.H., Ihlemann N., Jørgensen T.H. (2019). Five-Year Clinical and Echocardiographic Outcomes from the Nordic Aortic Valve Intervention (NOTION) Randomized Clinical Trial in Lower Surgical Risk Patients. Circulation.

[32] Søndergaard L., Ihlemann N., Capodanno D. (2019). Durability of Transcatheter and Surgical Bioprosthetic Aortic Valves in Patients at Lower Surgical Risk. J. Am. Coll. Cardiol..

[33] Zhang X.L., Zhang X.W., Lan R.F., Chen Z., Wang L., Xu W., Xu B. (2021). Long-term and Temporal Outcomes of Transcatheter Versus Surgical Aortic-valve Replacement in Severe Aortic Stenosis: A Meta-analysis. Ann Surg..

[34] Zajarias A., Cribier A.G. (2009). Outcomes and safety of percutaneous aortic valve replacement. J. Am. Coll. Cardiol..

[35] Generaux P., Head S.J., Hahn R., Daneault B., Kodali S., Williams M.R., van Mieghem N. (2013). Paravalvular leak after transcatheter aortic valve replacement: The new Achilles’ heel?. J. Am. Coll. Cardiol..

[36] Blanke P., Siepe M., Reinöhl J., Zehender M., Beyersdorf F., Schlensak C., Langer M., Pache G. (2010). Assessment of aortic annulus dimensions for Edwards SAPIEN Transapical Heart Valve implantation by computed tomography: Calculating average diameter using a virtual ring method. Eur. J. Cardiothorac. Surg..

[37] Détaint D., Lepage L., Himbert D., Brochet E., Messika-Zeitoun D., Iung B., Vahanian A. (2009). Determinants of significant paravalvular regurgitation after transcatheter aortic valve: Implantation impact of device and annulus discongruence. JACC Cardiovasc. Interv..

[38] Kappetein A.P., Head S.J., Généreux P. (2012). Updated standardized endpoint definitions for transcatheter aortic valve implantation: The Valve Academic Research Consortium-2 consensus document. J. Am. Coll. Cardiol..

[39] Nappi F., Mazzocchi L., Avtaar Singh S.S. (2018). Complementary Role of the Computed Biomodelling through Finite Element Analysis and Computed Tomography for Diagnosis of Transcatheter Heart Valve Thrombosis. Biomed Res Int..

[40] Morganti S., Conti M., Aiello M. (2014). Simulation of transcatheter aortic valve implantation through patient- specific finite element analysis: Two clinical cases. J. Biomech..

[41] Morganti S., Brambilla N., Petronio A.S. (2016). Prediction of patient-specific post-operative outcomes of TAVI procedure: The impact of the positioning strategy on valve performance. J. Biomech..

[42] Bianchi M., Marom G., Ghosh R.P., Rotman O.M., Parikh P., Gruberg L., Bluestein D. (2019). Patient-specific simulation of transcatheter aortic valve replacement: Impact of deployment options on paravalvular leakage. Biomech. Model Mechanobiol..

[43] Wang Q., Sirois E., Sun W. (2012). Patient-specific modeling of biomechanical interaction in transcatheter aortic valve deployment. J. Biomech..

[44] De Jaegere P. (2016). Patient-specific computer modeling to predict aortic regurgitation after transcatheter aortic valve replacement. JACC Cardiovasc. Interv..

[45] Capelli C. (2012). Patient-specific simulations of transcatheter aortic valve stent implantation. Med. Biol. Eng. Comput..

[46] Bosmans B., Famaey N., Verhoelst E., Bosmans J., Vander Sloten J. (2016). A validated methodology for patient specific computational modeling of self-expandable transcatheter aortic valve implantation. J. Biomech..

[47] Bianchi M., Marom G., Ghosh R.P., Fernandez H.A., Taylor J.R., Slepian M.J., Bluestein D. (2016). Effect of balloon-expandable transcatheter aortic valve replacement positioning: A patient-specific numerical model. Artif Organs..

[48] Bosi G.M., Capelli C., Hong Cheang M., Delahunty N., Mullen M., Taylor A.M., Schievano S. (2018). Population-specific material properties of the implantation site for transcatheter aortic valve replacement finite element simulations. J. Biomech..

[49] Dowling C., Firoozi S., Brecker S.J. (2020). First-in-Human Experience with Patient-Specific Computer Simulation of TAVR in Bicuspid Aortic Valve Morphology. JACC Cardiovasc. Interv..

[50] Nappi F., Mazzocchi L., Spadaccio C., Attias D., Timofeva I., Macron L., Iervolino A., Morganti S., Auricchio F. (2021). CoreValve vs. Sapien 3 Transcatheter Aortic Valve Replacement: A Finite Element Analysis Study. Bioengineering.

[51] Spadaccio C., Mazzocchi L., Timofeva I., Macron L., De Cecco C.N., Morganti S., Auricchio F., Nappi F. (2020). Bioengineering Case Study to Evaluate Complications of Adverse Anatomy of Aortic Root in Transcatheter Aortic Valve Replacement: Combining Biomechanical Modelling with CT imaging. Bioengineering.

[52] Nappi F., Mazzocchi L., Timofeva I., Macron L., Morganti S., Avtaar Singh S.S., Attias D., Congedo A., Auricchio F. (2020). A Finite Element Analysis Study from 3D CT to Predict Transcatheter Heart Valve Thrombosis. Diagnostics.

[53] Bonhoeffer P., Boudjemline Y., Saliba Z. (2000). Transcatheter implantation of a bovine valve in pulmonary position: A lamb study. Circulation.

[54] Nappi F., Nenna A., Larobina D., Carotenuto A.R., Jarraya M., Spadaccio C., Fraldi M., Chello M., Acar C., Carrel T. (2018). Simulating the ideal geometrical and biomechanical parameters of the pulmonary autograft to prevent failure in the Ross operation. Interact. Cardiovasc. Thorac. Surg..

[55] Nappi F., Nenna A., Lemmo F., Chello M., Chachques J.C., Acar C., Larobina D. (2020). Finite Element Analysis Investigate Pulmonary Autograft Root and Leaflet Stresses to Understand Late Durability of Ross Operation. Biomimetics.

[56] Xuan Y., Krishnan K., Ye J., Dvir D., Guccione J.M., Ge L. (2017). Stent and leaflet stresses in a 26-mm first-generation balloon-expandable transcatheter aortic valve. J. Thorac. Cardiovasc. Surg..

[57] Li K., Sun W. (2010). Simulated thin pericardial bioprosthetic valve leaflet deformation under static pressure-only loading conditions: Implications for percutaneous valves. Ann. Biomed. Eng..

[58] Nappi F., Carotenuto A.R., Cutolo A. (2016). Compliance mismatch and compressive wall stresses drive anomalous remodelling of pulmonary trunks reinforced with Dacron grafts. J. Mech. Behav. Biomed. Mater..

[59] Nappi F., Fraldi M., Spadaccio C. (2016). Biomechanics drive histological wall remodeling of neoaortic root: A mathematical model to study the expression levels of ki 67, metalloprotease, and apoptosis transition. J. Biomed. Mater. Res. A..

[60] Nappi F., Attias D., Avtaar Singh S.S. (2019). Finite element analysis applied to the transcatheter mitral valve therapy: Studying the present, imagining the future. J. Thorac. Cardiovasc. Surg..

[61] Nappi F., Spadaccio C., Al-Attar N., Acar C. (2015). The Ross procedure at the crossroads: Lessons from biology: Is Dr Ross’s dream concluded?. Int. J. Cardiol..

[62] Nataf P., Guettier C., Bourbon A., Nappi F., Lima L., Dorent R., Pavie A., Gandjbakhch I. (1996). Influence of arterial allograft preparation techniques on chronic vascular rejection: A histological study. Transplant Proc..

[63] Nappi F., Carotenuto A.R., Avtaar Singh S.S., Mihos C., Fraldi M. (2019). Euler’s Elastica-Based Biomechanics of the Papillary Muscle Approximation in Ischemic Mitral Valve Regurgitation: A Simple 2D Analytical Model. Materials.

[64] Rama A., Nappi F., Praschker B.G., Gandjbakhch I. (2008). Papillary muscle approximation for ischemic mitral valve regurgitation. J. Card Surg..

[65] Spadaccio C., Nappi F., De Marco F. (2017). Implantation of a Poly-L-Lactide GCSF-Functionalized Scaffold in a Model of Chronic Myocardial Infarction. J. Cardiovasc. Transl. Res..

[66] Spadaccio C., Nappi F., De Marco F. (2016). Preliminary In Vivo Evaluation of a Hybrid Armored Vascular Graft Combining Electrospinning and Additive Manufacturing Techniques. Drug Target Insights.

[67] Nappi F., Carotenuto A.R., Di Vito D., Spadaccio C., Acar C., Fraldi M. (2016). Stress-shielding, growth and remodeling of pulmonary artery reinforced with copolymer scaffold and transposed into aortic position. Biomech. Model Mechanobiol..

[68] Jamari J., Ammarullah M.I., Santoso G., Sugiharto S., Supriyono T., Prakoso A.T., Basri H., van der Heide E. (2022). Computational Contact Pressure Prediction of CoCrMo, SS 316L and Ti6Al4V Femoral Head against UHMWPE Acetabular Cup under Gait Cycle. J. Funct. Biomater..

[69] Smuts A.N., Blaine D.C., Scheffer C., Weich H., Doubell A.F., Dellimore K.H. (2011). Application of finite element analysis to the design of tissue leaflets for a percutaneous aortic valve. J. Mech. Behav. Biomed. Mater..

[70] Sun W., Li K., Sirois E. (2010). Simulated elliptical bioprosthetic valve deformation: Implications for asymmetric transcatheter valve deployment. J. Biomech..

[71] Auricchio F., Conti M., Morganti S., Reali A. (2014). Simulation of transcatheter aortic valve implantation: A patient-specific finite element approach. Comput. Methods Biomech. Biomed. Engin..

[72] Martin C., Sun W. (2015). Comparison of transcatheter aortic valve and surgical bioprosthetic valve durability: A fatigue simulation study. J. Biomech..

[73] Ammarullah M.I., Afif I.Y., Maula M.I., Winarni T.I., Tauviqirrahman M., Akbar I., Basri H., van der Heide E., Jamari J. (2021). Tresca Stress Simulation of Metal-on-Metal Total Hip Arthroplasty during Normal Walking Activity. Materials.

[74] Raby J., Newton J.D., Dawkins S., Lewis A.J.M. (2021). Cardiovascular magnetic resonance facilitates entirely contrast-free transcatheter aortic valve implantation: Case report. Eur. Heart J. Case Rep..

[75] Bittner D.O., Arnold M., Klinghammer L., Schuhbaeck A., Hell M.M., Muschiol G., Gauss S., Lell M., Uder M., Hoffmann U. (2016). Contrast volume reduction using third generation dual source computed tomography for the evaluation of patients prior to transcatheter aortic valve implantation. Eur. Radiol..

[76] Yushkevich P.A., Piven J., Hazlett H.C., Smith R.G., Ho S., Gee J.C., Gerig G. (2006). User-guided 3D active contour segmentation of anatomical structures: Significantly improved efficiency and reliability. Neuroimage.

[77] Antiga L., Piccinelli M., Botti L., Ene Iordache B., Remuzzi A., Steinman D.A. (2008). An image-based modeling framework for patient-specific computational hemodynamics. Med. Biol. Eng. Comput..

[78] Piccinelli M., Veneziani A., Steinman D.A., Remuzzi A., Antiga L. (2009). A framework for geometric analysis of vascular structures: Application to cerebral aneurysms. IEEE Trans. Med. Imaging.

[79] Marchandise E., Geuzaine C., Remacle J.F. (2013). Cardiovascular and lung mesh generation based on centerlines. Int. J. Numer. Method Biomed. Eng..

[80] Dillard S.I., Mousel J.A., Shrestha L., Raghavan M.L., Vigmostad S.C. (2014). From medical images to flow computations without user-generated meshes. Int. J. Numer. Method Biomed. Eng..

[81] Xiong F.L., Goetz W.A., Chong C.K., Chua Y.L., Pfeifer S., Wintermantel E., Yeo J.H. (2010). Finite element investigation of stentless pericardial aortic valves: Relevance of leaflet geometry. Ann Biomed Eng..

[82] Stradins P., Lacis R., Ozolanta I., Purina B., Ose V., Feldmane L., Kasyanov V. (2004). Comparison of biomechanical and structural properties between human aortic and pulmonary valve. Eur. J. Cardiothorac Surg..

[83] Gnyaneshwar R., Kumar R.K., Balakrishnan K.R. (2002). Dynamic analysis of the aortic valve using a finite element model. Ann. Thorac. Surg..

[84] Selvadurai A.P.S. (2006). Deflections of a rubber membrane. J. Mech. Phys. Solids.

[85] Yeoh O.H. (1993). Some forms of the strain energy function for rubber. Rubber Chem. Technol..

[86] Auricchio F., Ferrara A., Morganti S. (2012). Comparison and critical analysis of invariant-based models with respect to their ability in fitting human aortic valve data. Ann. Solid Struct. Mech..

[87] Hanlon J.G., Suggit R.W., Love J.W. (1999). Pre-use intraoperative testing of autologous tissue for valvular surgery: A proof-of-concept study. J. Heart Valve Dis..

[88] Lee J.M., Haberer S.A., Boughner D.R. (1989). The bovine pericardial xenograft: I. Effect of fixation in aldehydes without constraint on the tensile viscoelastic properties of bovine pericardium. J. Biomed. Mater Res..

[89] Trowbridge E.A., Black M.M., Daniel C.L. (2011). The mechanical response of glutaraldehyde fixed bovine pericardium to uniaxial load. J. Mater. Sci..

[90] Willson A.B., Rodés-Cabau J., Wood D.A., Leipsic J., Cheung A., Toggweiler S., Binder R.K., Freeman M. (2012). Transcatheter aortic valve replacement with the St. Jude Medical Portico valve: First-in-human experience. J. Am. Coll. Cardiol..

[91] Möllmann H., Diemert P., Grube E., Baldus S., Kempfert J., Abizaid A. (2013). Symetis ACURATE TF™ aortic bioprosthesis. EuroIntervention.

[92] Meredith I.T., Hood K.L., Haratani N., Allocco D.J., Dawkins K.D. (2012). Boston Scientific Lotus valve. EuroIntervention.

[93] Feldman T.E., Reardon M.J., Rajagopal V. (2018). Effect of Mechanically Expanded vs Self-Expanding Transcatheter Aortic Valve Replacement on Mortality and Major Adverse Clinical Events in High-Risk Patients Wit50,52h Aortic Stenosis: The REPRISE III Randomized Clinical Trial. JAMA.

[94] Wiggers C.J. (1952). Circulatory Dynamics: Physiological Studies. JAMA.

[95] Delgado V., Ng A.C., van de Veire N.R., van der Kley F., Schuijf J.D., Tops L.F. (2010). Transcatheter aortic valve implantation: Role of multi-detector row computed tomography to evaluate prosthesis positioning and deployment in relation to valve function. Eur. Heart J..

[96] Santos N., de Agustín J.A., Almería C., Gonçalves A., Marcos-Alberca P., Fernández-Golfín C. (2012). Prosthesis/annulus discongruence assessed by three-dimensional transoesophageal echocardiography: A predictor of significant paravalvular aortic regurgitation after transcatheter aortic valve implantation. Eur. Heart J. Cardiovasc. Imaging.

[97] Pontone G., Andreini D., Bartorelli A.L., Bertella E., Cortinovis S., Mushtaq S., Annoni A. (2012). Aortic annulus area assessment by multidetector computed tomography for predicting paravalvular regurgitation in patients undergoing balloon-expandable transcatheter aortic valve implantation: A comparison with transthoracic and transoesophageal echocardiography. Am. Heart J..

[98] Katsanos S., Ewe S.H., Debonnaire P., van der Kley F., de Weger A., Palmen M., Scholte A.J. (2013). Multidetector row computed tomography parameters associated with paravalvular regurgitation after transcatheter aortic valve implantation. Am. J. Cardiol..

[99] Madukauwa-David I.D., Midha P.A., Sharma R., McLain K., Mitra R., Crawford K., Yoon S.H. (2019). Characterization of aortic root geometry in transcatheter aortic valve replacement patients. Catheter. Cardiovasc. Interv..

[100] Feuchtner G., Plank F., Bartel T., Mueller S., Leipsic J., Schachner T., Müller L. (2013). Prediction of paravalvular regurgitation after transcatheter aortic valve implantation by computed tomography: Value of aortic valve and annular calcification. Ann. Thorac. Surg..

[101] Eker A., Sozzi F.B., Civaia F., Bourlon F. (2012). Aortic annulus rupture during transcatheter aortic valve implantation: Safe aortic root replacement. Eur. J. Cardiothorac. Surg..

[102] Wang Q., Kodali S., Primiano C., Sun W. (2015). Simulations of transcatheter aortic valve implantation: Implications for aortic root rupture. Biomech. Model Mechanobiol..

[103] Auricchio F., Conti M., Morganti S., Totaro P. (2011). A computational tool to support pre-operative planning of stentless aortic valve implant. Med. Eng. Phys..

[104] Auricchio F., Conti M., Ferrara A., Morganti S., Reali A. (2012). Patient-specific simulation of a stentless aortic valve implant: The impact of fibres on leaflet performance. Comput. Methods Biomech. Biomed. Eng..

[105] Morlacchi S., Colleoni S.G., Cárdenes R., Chiastra C., Diez J.L., Larrabide I., Migliavacca F. (2013). Patient-specific simulations of stenting procedures in coronary bifurcations: Two clinical cases. Med. Eng. Phys..

[106] Grover A., Gorman K., Dall T.M., Jonas R., Lytle B., Shemin R. (2009). Shortage of Cardiothoracic Surgeons Is Likely by 2020. Circulation.

[107] Kuhn T.S. (1963). The Structure of Scientific Revolutions. Am. Hist. Rev..

[108] Holmes D.R., Firth B.G., Wood D.L. (2004). Paradigm shifts in cardiovascular medicine. J. Am. Coll. Cardiol..

[109] Sacks C.A., Jarcho J.A., Curfman G.D. (2014). Paradigm shifts in heart-failure therapy—A timeline. New Engl. J. Med..

[110] Kanwar A., Thaden J.J., Nkomo V.T. (2018). Management of patients with aortic valve stenosis. Mayo Clin. Proc..

[111] Pilgrim T., Windecker S. (2015). Transcatheter aortic valve replacement: Lessons gained from extreme-risk patients. J. Am. Coll. Cardiol..

[112] Bagur R., Rodés-Cabau J., Gurvitch R., Dumont É., Velianou J.L., Manazzoni J., Toggweiler S. (2012). Need for permanent pacemaker as a complication of transcatheter aortic valve implantation and surgical aortic valve replacement in elderly patients with severe aortic stenosis and similar baseline electrocardiographic findings. JACC Cardiovasc. Interv..

[113] Van der Boon R.M., Nuis R.J., Van Mieghem N.M., Jordaens L., Rodés-Cabau J., van Domburg R.T., Serruys P.W. (2012). New conduction abnormalities after TAVI—frequency and causes. Nat. Rev. Cardiol. Nat. Rev. Cardiol..

[114] Ribeiro H.B. (2013). Coronary obstruction following transcatheter aortic valve implantation: A systematic review. JACC Cardiovasc. Interv..

[115] Scotten L.N., Siegel R. (2014). Thrombogenic potential of transcatheter aortic valve implantation with trivial paravalvular leakage. Ann. Transl. Med..

[116] Maisano F., Taramasso M., Nietlispach F. (2015). Prognostic influence of paravalvular leak following TAVI: Is aortic regurgitation an active incremental risk factor or just a mere indicator?. Eur. Heart J..

[117] Gilbert O.N. (2018). Comparison of paravalvular aortic leak characteristics in the Medtronic CoreValve versus Edwards Sapien Valve: Paravalvular aortic leak characteristics. Catheter. Cardiovasc. Interv..

[118] Still S., Szerlip M., Mack M. (2018). TAVR Vs. SAVR in intermediate-risk patients: What influences our choice of therapy. Curr. Cardiol. Rep..

[119] Pibarot P., Hahn R.T., Weissman N.J., Monaghan M.J. (2015). Assessment of paravalvular regurgitation following TAVR: A proposal of unifying grading scheme. JACC Cardiovasc. Imag..

[120] Hatoum H., Yousefi A., Lilly S., Maureira P., Crestanello J., Dasi L.P. (2018). An in-vitro evaluation of turbulence after transcatheter aortic valve implantation. J. Thorac. Cardiovasc. Surg..

[121] Abdelghani M., Soliman O.I.I., Schultz C., Vahanian A., Serruys P.W. (2016). Adjudicating paravalvular leaks of transcatheter aortic valves: A critical appraisal. Eur. Heart J..

[122] Eggebrecht H., Doss M., Schmermund A., Nowak B., Krissel J., Voigtländer T. (2012). Interventional options for severe aortic regurgitation after transcatheter aortic valve implantation: Balloons, snares, valve-in-valve. Clin. Res. Cardiol..

[123] Dvir D. (2012). Multicenter evaluation of Edwards SAPIEN positioning during transcatheter aortic valve implantation with correlates for device movement during final deployment. JACC Cardiovasc. Interv..

[124] Nombela-Franco L. (2012). Predictive factors, efficacy, and safety of balloon post-dilation after transcatheter aortic valve implantation with a balloon-expandable valve. JACC Cardiovasc. Interv..

[125] Takagi K. (2011). Predictors of moderate-to-severe paravalvular aortic regurgitation immediately after CoreValve implantation and the impact of postdilatation. Catheter. Cardiovasc. Interv..

[126] McGee O.M., Gunning P.S., McNamara A., McNamara L.M. (2018). The impact of implantation depth of the Lotus™ valve on mechanical stress in close proximity to the bundle of His. Biomech Model Mechanobiol..

[127] Sturla F. (2016). Impact of different aortic valve calcification patterns on the outcome of Transcatheter Aortic Valve Implantation: A finite element study. J. Biomech..

[128] Schultz C. (2016). Patient-specific image-based computer simulation for the prediction of valve morphology and calcium displacement after TAVI with the Medtronic CoreValve and the Edwards SAPIEN valve. EuroIntervention.

[129] Chang J., Rong-Hui L., Sheng-Ping Z., Li-Zhen W., Yu-Bo F. (2018). Effect of stent designs on the paravalvular regurgitation of transcatheter aortic valve implantation. Int. J. Comput. Methods.

[130] Mao W., Wang Q., Kodali S., Sun W. (2018). Numerical parametric study of paravalvular leak following a transcatheter aortic valve deployment into a patient-specific aortic root. J. Biomech. Eng..

[131] Vahidkhah K., Azadani A.N. (2017). Supra-annular Valve-in-Valve implantation reduces blood stasis on the transcatheter aortic valve leaflets. J. Biomech..

[132] Latib A., Naganuma T., Abdel-Wahab M. (2015). Treatment and clinical outcomes of transcatheter heart valve thrombosis. Circ. Cardiovasc. Interv..

[133] Stortecky S., Windecker S. (2012). Stroke: An infrequent but devastating complication in cardiovascular interventions. Circulation.

[134] Leetmaa T., Hansson N.C., Leipsic J. (2015). Early aortic transcatheter heart valve thrombosis: Diagnostic value of contrast-enhanced multidetector computed tomography. Circ. Cardiovasc. Interv..

[135] Hansson N.C., Grove E.L., Andersen H.R. (2016). Transcatheter aortic heart valve thrombosis: Incidence, predisposing factors, and clinical implications. J. Am. Coll. Cardiol..

[136] Wolberg A.S., Aleman M.M., Leiderman K. (2012). Procoagulant activity in hemostasis and thrombosis: Virchow’s triad revisited. Anesth. Analg..

[137] Turbill P., Beugeling T., Poot A.A. (1996). Proteins involved in the Vroman effect during exposure of human blood plasma to glass and polyethylene. Biomaterials.

[138] Noble S., Asgar A., Cartier R. (2009). Anatomopathological analysis after CoreValve Revalving system implantation. EuroIntervention.

[139] Makkar R.R., Fontana G., Jilaihawi H. (2015). Possible subclinical leaflet thrombosis in bioprosthetic aortic valves. N. Engl. J. Med..

[140] Chakravarty T., Søndergaard L., Friedman J., RESOLVE, SAVORY Investigators (2017). Subclinical leaflet thrombosis in surgical and transcatheter bioprosthetic aortic valves: An observational study. Lancet.

[141] Pache G., Schoechlin S., Blanke P. (2016). Early hypo-attenuated leaflet thickening in balloon-expandable transcatheter aortic heart valves. Eur. Heart J..

[142] Vollema E.M., Kong W.K.F., Katsanos S. (2017). Transcatheter aortic valve thrombosis: The relation between hypo-attenuated leaflet thickening, abnormal valve haemodynamics, and stroke. Eur. Heart J..

[143] Nührenberg T.G., Hromek J., Kille A. (2019). Impact of On-Clopidogrel Platelet Reactivity on Incidence of Hypoattenuated Leaflet Thickening After Transcatheter Aortic Valve Replacement. JACC Cardiovasc. Interv..

[144] De Backer O., Dangas G.D., Jilaihawi H. (2020). GALILEO-4D Investigators. Reduced Leaflet Motion after Transcatheter Aortic-Valve Replacement. N. Engl. J. Med..

[145] Khalique O.K., Hahn R.T., Gada H. (2014). Quantity and location of aortic valve complex calcification predicts severity and location of paravalvular regurgitation and frequency of post-dilation after balloon-expandable transcatheter aortic valve replacement. JACC Cardiovasc. Interv..

[146] Couture E.L., Lepage S., Masson J.-B., Daneault B. (2017). Very late transcatheter heart valve thrombosis. World J. Cardiol..

[147] Lancellotti P., Pibarot P., Chambers J. (2016). Recommendations for the imaging assessment of prosthetic heart valves: A report from the European Association of Cardiovascular Imaging endorsed by the Chinese Society of Echocardiography, the Inter-American Society of Echocardiography, and the Brazilian Department of Cardiovascular Imaging. Eur. Heart J. Cardiovasc. Imaging.

[148] Capodanno D., Petronio A.S., Prendergast B. (2017). Standardized definitions of structural deterioration and valve failure in assessing long-term durability of transcatheter and surgical aortic bioprosthetic valves: A consensus statement from the European Association of Percutaneous Cardiovascular Interventions (EAPCI) endorsed by the European Society of Cardiology (ESC) and the European Association for Cardio-Thoracic Surgery (EACTS). Eur. J. Cardiothorac. Surg..

[149] Zilberszac R., Gabriel H., Schemper M. (2013). Outcome of combined stenotic and regurgitant aortic valve disease. J. Am. Coll. Cardiol..

[150] Masters R.G., Walley V.M., Pipe A.L. (1995). Long-term experience with the Ionescu-Shiley pericardial valve. Ann. Thorac. Surg..

[151] Puvimanasinghe J.P., Steyerberg E.W., Takkenberg J.J. (2001). Prognosis after aortic valve replacement with a bioprosthesis: Predictions based on meta-analysis and microsimulation. Circulation.

[152] Wang M., Furnary A.P., Li H.F. (2017). Bioprosthetic aortic valve durability: A meta-regression of published studies. Ann. Thorac. Surg..

[153] Foroutan F., Guyatt G.H., O’Brien K. (2016). Prognosis after surgical replacement with a bioprosthetic aortic valve in patients with severe symptomatic aortic stenosis: Systematic review of observational studies. BMJ.

[154] Nappi F., Nenna A., Petitti T., Spadaccio C., Gambardella I., Lusini M., Chello M., Acar C. (2018). Long-term outcome of cryopreserved allograft for aortic valve replacement. J. Thorac. Cardiovasc. Surg..

[155] Fukushima S., Tesar P.J., Pearse B. (2014). Long-term clinical outcomes after aortic valve replacement using cryopreserved aortic allograft. J. Thorac. Cardiovasc. Surg..

[156] Arabkhani B., Bekkers J.A., Andrinopoulou E.R. (2016). Allografts in aortic position: Insights from a 27-year, single-center prospective study. J. Thorac. Cardiovasc. Surg..

[157] David T.E., Feindel C.M., Bos J. (2008). Aortic valve replacement with Toronto SPV bioprosthesis: Optimal patient survival but suboptimal valve durability. J. Thorac. Cardiovasc. Surg..

[158] Schaefer A., Dickow J., Schoen G. (2018). Stentless vs stented bioprosthesis for aortic valve replacement: A case matched comparison of long-term follow-up and subgroup analysis of patients with native valve endocarditis. PLoS ONE.

[159] Nishida T., Tominaga R. (2013). A look at recent improvements in the durability of tissue valves. Gen. Thorac. Cardiovasc. Surg..

[160] Garrido-Olivares L., Maganti M., Armstrong S., David T. (2011). Aortic valve replacement with Hancock II bioprosthesis with and without replacement of the ascending aorta. Ann. Thorac. Surg..

[161] David T.E., Armstrong S., Maganti M. (2010). Hancock II bioprosthesis for aortic valve replacement: The gold standard of bioprosthetic valves durability?. Ann. Thorac. Surg..

[162] Glaser N., Franco-Cereceda A., Sartipy U. (2014). Late survival after aortic valve replacement with the perimount versus the mosaic bioprosthesis. Ann. Thorac. Surg..

[163] Bourguignon T., Bouquiaux-Stablo A.L., Candolfi P. (2015). Very long-term outcomes of the Carpentier-Edwards Perimount valve in aortic position. Ann. Thorac. Surg..

[164] Johnston D.R., Soltesz E.G., Vakil N. (2015). Long-term durability of bioprosthetic aortic valves: Implications from 12,569 implants. Ann. Thorac. Surg..

[165] Senage T., Le Tourneau T., Foucher Y. (2014). Early structural valve deterioration of Mitroflow aortic bioprosthesis: Mode, incidence, and impact on outcome in a large cohort of patients. Circulation.

[166] Goldman S., Cheung A., Bavaria J.E. (2017). Midterm, multicenter clinical and hemodynamic results for the Trifecta aortic pericardial valve. J. Thorac. Cardiovasc. Surg..

[167] Kalra A., Rehman H., Ramchandani M. (2017). Early Trifecta valve failure: Report of a cluster of cases from a tertiary care referral center. J. Thorac. Cardiovasc. Surg..

[168] Fischlein T., Meuris B., Hakim-Meibodi K. (2016). The sutureless aortic valve at 1 year: A large multicenter cohort study. J. Thorac. Cardiovasc. Surg..

[169] Kocher A.A., Laufer G., Haverich A. (2013). One-year outcomes of the Surgical Treatment of Aortic Stenosis With a Next Generation Surgical Aortic Valve (TRITON) trial: A prospective multicenter study of rapid-deployment aortic valve replacement with the EDWARDS INTUITY Valve System. J. Thorac. Cardiovasc. Surg..

[170] Durand E., Tron C., Eltchaninoff H. (2015). Emergency Transcatheter Aortic Valve Implantation for Acute and Early Failure of Sutureless Perceval Aortic Valve. Can. J. Cardiol..

[171] Tseng E., Wisneski A., Azadani A., Ge L. (2013). Engineering perspective on transcatheter aortic valve implantation. Interv. Cardiol..

[172] Sun W., Abad A., Sacks M.S. (2005). Simulated bioprosthetic heart valve deformation under quasi-static loading. J. Biomech. Eng..

[173] Alavi S.H., Groves E.M., Kheradvar A. (2014). The effects of transcatheter valve crimping on pericardial leaflets. Ann. Thorac. Surg..

